# A scoping review of cohort studies assessing traditional Chinese medicine interventions

**DOI:** 10.1186/s12906-020-03150-9

**Published:** 2020-11-23

**Authors:** Yuting Duan, Zhirui Xu, Jingjing Deng, Yanjia Lin, Yan Zheng, Juexuan Chen, Xiaoyu Tang, Xuan Zhang, Chunzhi Tang, Jiangxia Miao, Zhaoxiang Bian

**Affiliations:** 1 Hong Kong Chinese Medicine Clinical Study Center, School of Chinese Medicine, Hong Kong Baptist University, 3/F, Jockey Club School of Chinese Medicine Building, 7 Baptist University Road, Kowloon Tong, Hong Kong, SAR China; 2 Chinese EQUATOR Center, Hong Kong, SAR China; 3 Medical College of Acu-Moxi and Rehabilitation, Guangzhou University of Chinese Medicine, Guangzhou, China; 4 Puning Traditional Chinese Medicine Hospital, Jieyang, China; 5 American Academy of Acupuncture and Oriental Medicine, Roseville, Minnesota USA; 6 School of Chinese medicine, The Chinese University of Hong Kong, Hong Kong, SAR China

**Keywords:** Scoping review, Cohort studies, Traditional Chinese medicine, Reporting quality, Evidence-based medicine

## Abstract

**Backgrounds:**

Identifying topics and assessing the reporting quality of Traditional Chinese Medicine (TCM) cohort studies.

**Methods:**

A scoping review of the literature was performed. A descriptive approach to summarize the core study characteristics was prepared, along with structured tables and figures to identify salient points of differences noted across studies. The reporting quality of TCM cohort studies was assessed according to the Strengthening the Reporting of Observational Studies in Epidemiology (STROBE)-cohort checklist.

**Results:**

A total of 199 TCM cohort studies were included. The largest number of TCM cohort studies was conducted in Mainland China (70.9%). The TCM cohort study was first published in 2003. The top three diseases studied were Acquired Immune Deficiency Syndrome (AIDS), Stroke, and Asthma. As for the intervention methods, Chinese herbal medicine formulas (60.3%), acupuncture (14.1%) and single herbs (12.6%) accounted for the majority, followed by moxibustion (4.0%) and qigong (2.0%). The overage sufficient reporting rate of included TCM cohort studies according to the STROBE-cohort checklist was 42.9%. Comparing with Chinese literature, the reporting rates of English literature in most items were higher.

**Conclusion:**

For the application of cohort studies to inform the effects of TCM interventions, the interventions assessed and conditions studied were diverse, the reporting quality was unsatisfied.

**Supplementary Information:**

The online version contains supplementary material available at 10.1186/s12906-020-03150-9.

## Backgrounds

Cohort study, as one of the classical epidemiological observational research methods, is widely used in clinical research, and its research results are second only to randomized controlled trials (RCT) in the evidence-based evaluation hierarchy of evidence. Compared with RCT, cohort studies can better reflect the effects of traditional Chinese medicine (TCM) interventions in the real diagnosis and treatment environment, have strong external authenticity [1], and can fully reflect the characteristics of individualized syndrome differentiation and treatment of TCM [2].

According to our previous research, the number of published domestic and foreign cohort studies in TCM has increased year by year. Meanwhile, cohort studies accounted the largest of the three main types of observational studies in TCM [3]. When cohort studies applied to the field of TCM, the TCM interventions would be considered as the exposure factors [4]. The topics of TCM cohort studies assessing TCM interventions mainly are efficacy evaluation [5–7], prognosis of diseases [8, 9] adverse events [10, 11], and economic evaluation [12, 13].

In 2007, the “Strengthening the Reporting of Observational Studies in Epidemiology”- STROBE statement [14] was published in the LANCET and other 7 peer-review journals. The statement contains a checklist of 22 items, including the title, abstract, introduction, methods, results, and discussion of the paper. Eighteen items apply to all three main observational study design, the remaining 4 items are dedicated to cohort, case control or cross-sectional study. The STROBE checklist is divided into STROBE-cohort, STROBE-case control and STROBE-cross sectional [15]. The STROBE statement is included in Enhancing the QUAlity and Transparency Of health Research (EQAUTOR) network and as a submission guide by multiple peer-reviewed journals [16, 17].

No review works has been done in topic analysis and reporting quality of TCM cohort studies so far. In the current study, we used a scoping review to establish the landscape of cohort studies with TCM interventions, to conduct the descriptive statistics of the objectives, clinical and methodologic characteristics, and to judge the current level of reporting transparency based upon criteria of the STROBE statement for TCM cohort studies. Findings from this review will be informative for researchers and stakeholders seeking to prioritize future topics for TCM cohort studies and improve the quality and transparency of TCM cohort studies.

## Methods

### Searching and screening literature

The following 8 databases: MEDLINE, Excerpta Medica Database (EMBASE), Cochrane Central Register of Controlled Trials (CENTRAL), the Allied and Complementary Medicine Database (AMED), Chinese Biomedical Literature Service System (CBM), China National Knowledge Infrastructure (CNKI), Wanfang Data and VIP Chinese Medical Journal Database were systematically searched for cohort studies in TCM that were published up to 18 October 2019. We searched the MEDLINE, EMBASE, CENTRAL, AMED through OVID platform. The search strategy is shown in Additional file 1. TCM cohort studies were screened according to the inclusion and exclusion criteria. The process of literature retrieval, screening and inclusion was completed by two researchers (ZRX, JJD) independently, and a third party senior researcher (YTD) would decide if there exist differences.

### Inclusion and exclusion criteria

We included cohort studies described TCM interventions, including Chinese herbal medicine formulas, single herbs, acupuncture, acupoint application, moxibustion, qigong, Tianjiu, pull-thread and medicated-thread. We excluded the modern physical stimulations and other complementary & alternative medicine therapies which not based on the TCM theories, for example, TENS (transcutaneous electrical nerve stimulation), tVNS (transcutaneous vagus nerve stimulation), rhythmical massage (anthroposophic medicine), homeopathy, other complementary and alternative medicine (aerobic exercise, dietary vitamins supplements, meditation and so on). For the publication language, we restricted to English and Chinese. For the type of cohort studies, we restricted the cohort studies assessing TCM interventions.

### Extracting basic characteristics

The basic information included the year of publication, the journal, the country and region where the research was carried out, the study design, the clinical conditions (the disease system and detailed disease), the main intervention and control method, the outcomes (whether used TCM related outcomes), the main research purpose, whether used medical databases, and whether obtained funding. The data extraction process was independently performed by two researchers (ZRX, JJD) to ensure the reliability of the data, and a third-party senior researcher (YTD) was introduced to decide when differences occurred.

### Pre-test and formal evaluation of the reporting quality

For assessing the reporting quality of TCM cohort studies, the STROBE-cohort checklist was used. Each item was scored according to four scenarios, “1” for “sufficient reported”, “2” for “insufficient reported”, “3” for “unreported”, and “4” for “not applicable”. Before the formal evaluation, 3 rounds of pre-tests were performed. The intra-class correlation coefficient (ICC) value was tested to judge the consistency of the evaluation results. ICC values> 0.75 were considered as good consistency. The average ICC values of three rounds pre-tests were 0.620, 0.785 and 0.829 respectively. The pre-tests reached good consistency before entering the formal evaluation. Two researchers (YTD, YJL) completed the formal evaluation independently. When disagreement happened, the judgment would be made by a third-party senior researcher (ZXB). During the evaluation process, attention should be paid to summarizing the inadequate reporting refer to each item of STROBE, and the possible reasons for the inadequate reporting were speculated.

### Data presentation

A descriptive approach to summarize the core study characteristics was prepared, along with structured tables and figures to identify salient points of differences noted across studies. Bar graphs were used to show the publication years, the categories of diseases, intervention methods, control methods, outcomes including TCM related indicators or not, research purposes and study designs. A heat map was generated to present the geographic distribution of published TCM cohort studies (based on the conducting countries or regions of included studies). The heat map was made by Tableau Desktop 2018.3.2 64bit (https://www.tableau.com/zh-cn/products/desktop). A word cloud was prepared to assess the relative frequencies with which different clinical conditions were studied in the set of included cohort studies. Percentage bar graphs were generated to present the proportions of included studies adequately matching individual items of the STROBE-cohort checklist. The comparison of the reporting rate in English and Chinese literature was presented in bar graph.

Our manuscript strictly follows the PRISMA guideline for scoping reviews (Additional file 4).

## Results

### Identified literature and the general characteristics

**Fig. 1:**
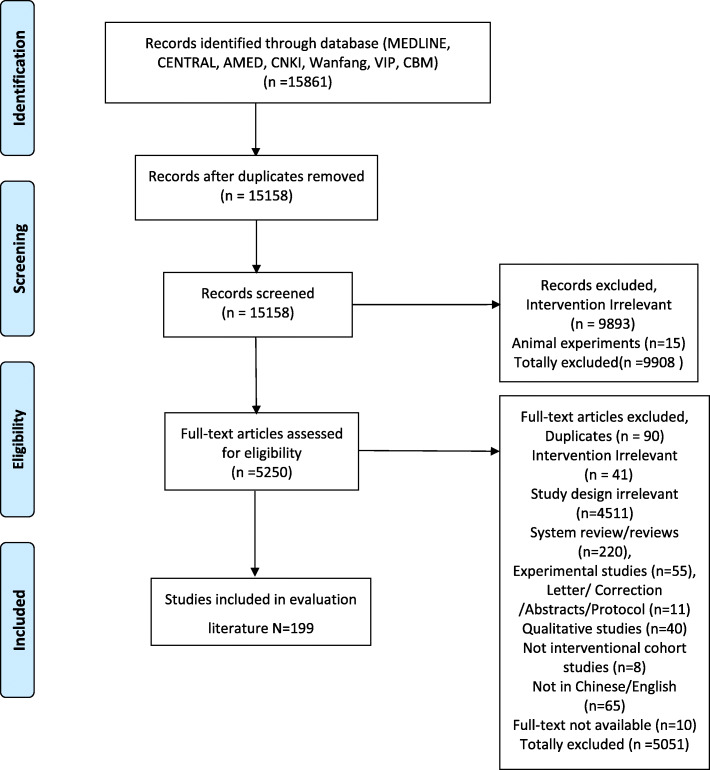
Flow chart

**Fig. 2:**
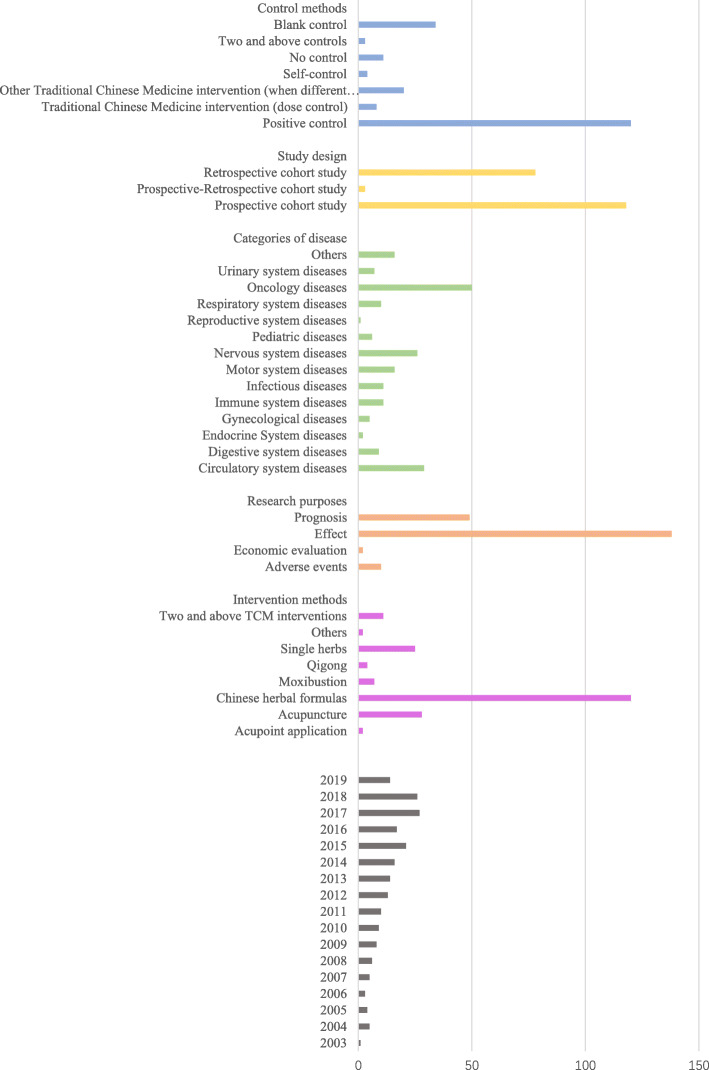
The basic characteristics of included TCM cohort studies

After searching and screening the literature, 199 TCM cohort studies were finally identified, which contained 74 English studies and 125 Chinese studies. The flow chart of the screening process was shown in Fig. 1. The overview of the included TCM cohort studies was presented in Additional file 2. The general characteristics of the included TCM cohort studies were shown in Fig. 2.

**Fig. 3:**
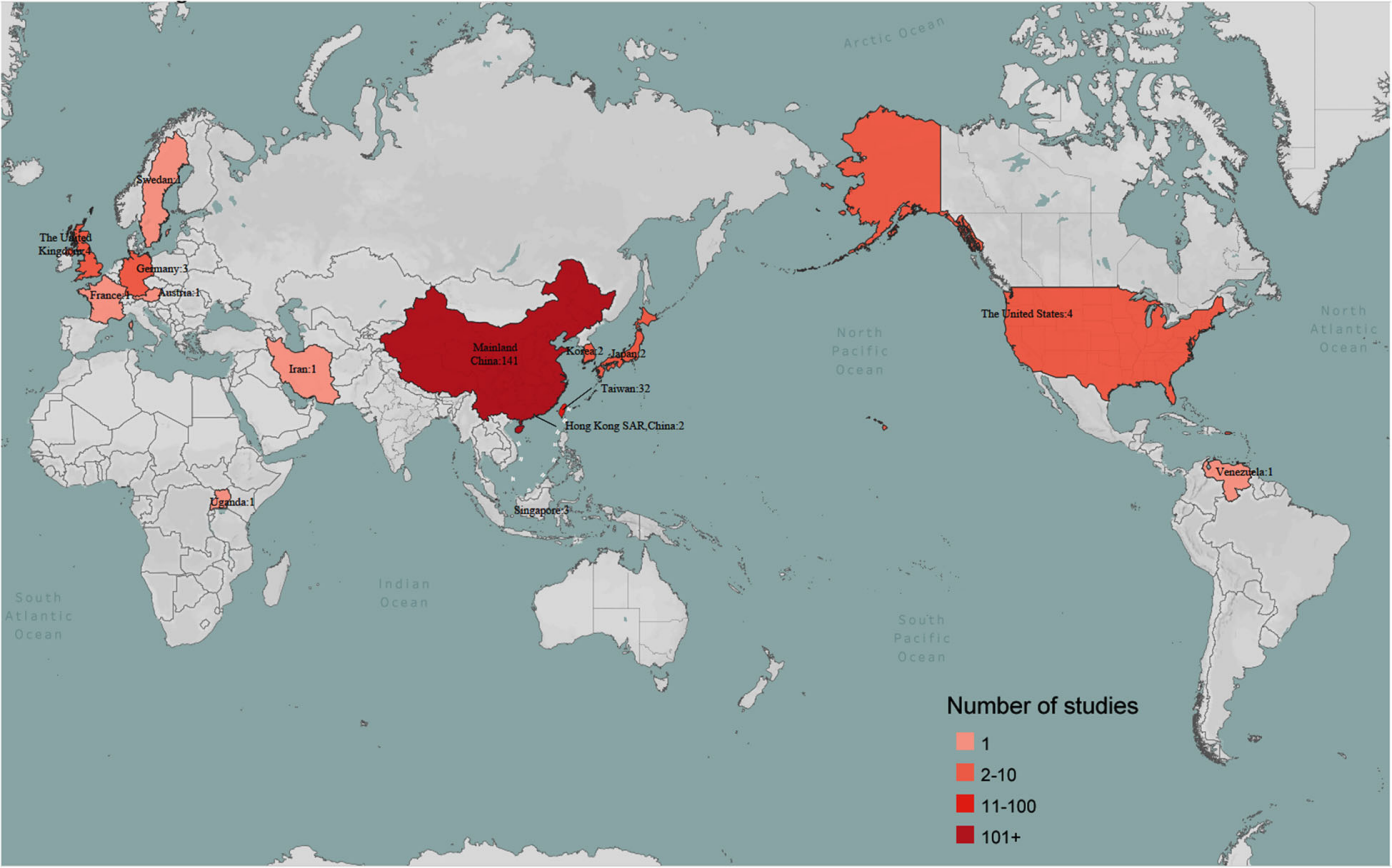
The distribution of conducted countries and regions of included TCM cohort studies

Judging from the changes in the publication year of the literature, the number of TCM cohort studies has been increasing year by year. The TCM cohort study was first published in 2003. As for the countries and regions where the research was conducted, most of the TCM cohort studies were conducted in Mainland China (70.9%) and Taiwan (16.1%). The rest were scatter performed in Hong Kong Special Administration Region (SAR), Singapore, Japan, South Korea, and Iran in Asia, the United States and Venezuela in America, and European Germany, France, Austria and Sweden, and Uganda in Africa. The detailed distribution of countries and regions conducting TCM cohort studies was shown in a heat map (Fig. 3).

### The clinical conditions, TCM interventions, control methods and outcomes

**Fig. 4:**
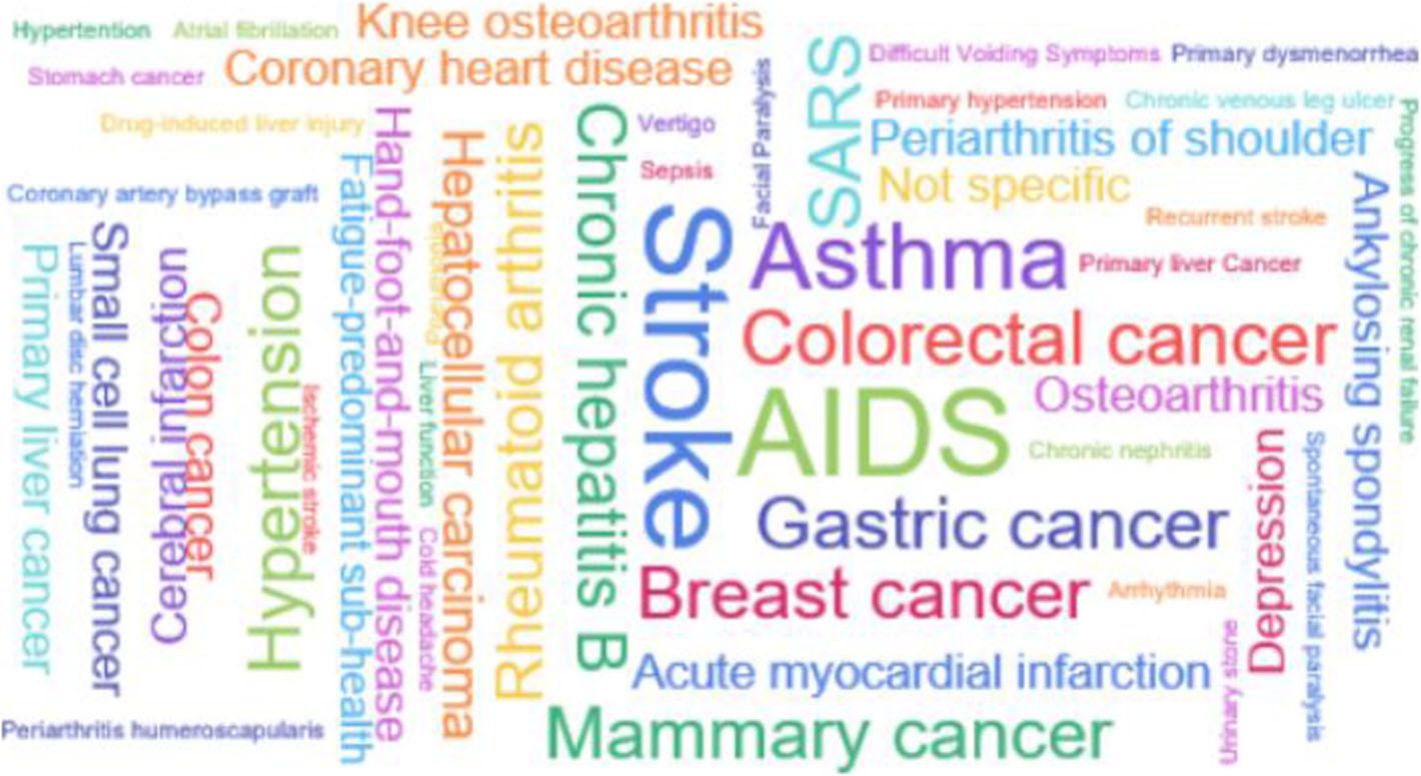
The frequency of specific diseases studied in the included TCM cohort studies

A diverse set of conditions was identified, the most common targeted conditions are oncology diseases (25.1%), circulatory system diseases (14.6%) and nervous system diseases (13.1%). A word cloud (Fig. 4) was prepared to show the frequency of research on specific diseases. The top three diseases studied were Acquired Immune Deficiency Syndrome (AIDS), Stroke and Asthma. As for the intervention methods, Chinese herbal medicine formulas (60.3%), acupuncture (14.1%) and single herbs (12.6%) accounted for the majority, followed by moxibustion (4.0%) and qigong (2.0%). The most commonly used control method was other TCM therapy (TCM interventions which differ from the intervention group), the other control methods like the positive control, blank control, dose control and self before and after control were account for a small proportion. In most studies using positive control, the intervention groups were based on the positive control plus Chinese medicine intervention measures (109/120, 90.8%). As for outcomes, there were only 23(11.6%) studies involving the TCM indicators, like TCM symptoms score.

### The research purpose and study design

The largest proportion of the research purpose in TCM cohort studies were mainly for efficacy (63.8%) and prognosis (24.6%) evaluation, followed by adverse events (5.0%) and economic evaluation (1.5%). From the summary of the study design, unidirectional cohort studies (98.5%) accounted for the vast majority, and there were more prospective studies (59.3%) than retrospective studies (39.2%). It was worth noting that there were 38 (19.1%) studies used local medical databases such as the National Health Insurance Research Database and the Taiwan Registry for Catastrophic Patients Database were used in the study.

### The reporting quality assessment

**Fig. 5:**
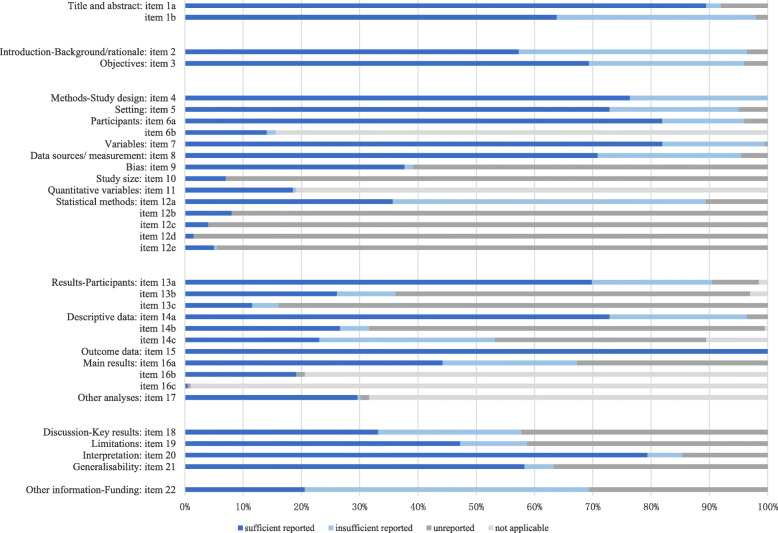
The reporting quality assessment of included TCM cohort studies

About the completeness of reporting, the proportion of included TCM cohort studies adequately reporting the 22 items of STROBE was summarized in Fig. 5 and Additional file 3. The overage sufficient reporting rate of included TCM cohort studies according to the STROBE-cohort checklist was 42.9%.

In the title, abstract, and background sections, the sufficient reporting rate was relatively high, exceeding 55%. For the whole checklist, it was important to point out that the non-applicability rate was more than 75% in 6b, 11, 16b, and 16c items.

In the methods section, items including 6b, 10, 11, 12b, 12c, 12d, and 12e were reported a low sufficient reporting rate (less than 20%). Items with a sufficient reporting rate between 20 and 50% were 9 and 12a. For item 10, the authors needed to explain how the study size was arrived at. However, only 0.07% of studies mentioned that. For item 12, the scores of the subitems represented the critical low reporting rate in the statistical method of TCM cohort studies. Concerned on item 9, the methods to control bias were only reported by 37.7% of studies.

In the results section, items 13c, 16b, and 16c which were sufficiently reported in included studies did not exceed 20%; 13b, 14b, 14c, and 16a were sufficiently reported for 20–50%. For the items 13b and 13c, the low reporting rate of them showed that the authors of TCM cohort studies failed in giving reasons for non-participation at each stage and the use of a flow diagram in describing the participants of studies. Regarding the items 14b and 14c, the low reporting rate of them illustrated that the insufficient transparency of missing data and follow-up time. For the item 16a, most studies (55.8%) showed a lack of reporting in confounder-adjusted estimates or reporting the confounder factors.

In the final discussion and other information section, the relatively poor reported items were 18, 19, and 22. They showed insufficient reporting in summary of results, limitations, and funding.

**Fig. 6:**
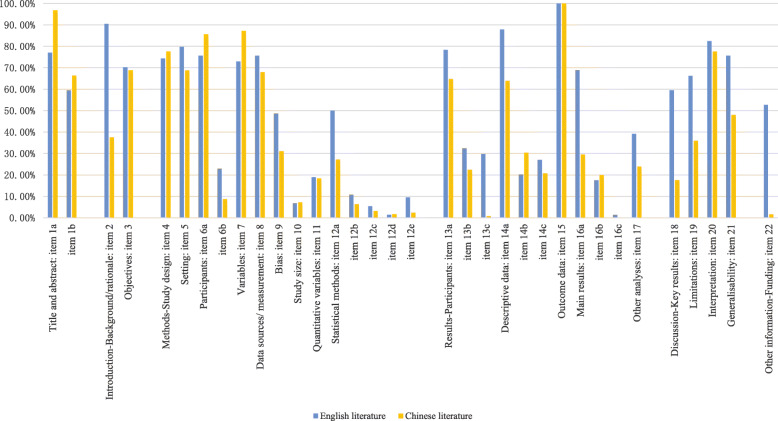
The comparison of the reporting rate of English and Chinese TCM cohort studies

We also compared the reporting rates of each item in the Chinese and English TCM cohort studies, the results were provided in Fig. 6. Given a difference of 20% as the criterion for the difference, the reporting rates of English literature of items 2, 12a, 14a, 16a, 18, 19, 21 and 22 were better than Chinese literature.

## Discussion

### Findings

The annual frequency of cohort studies involving TCM interventions has risen considerably in recent years, indicating the cohort studies have been a welcoming study design in TCM. While the largest number of TCM cohort studies included in this study was produced in mainland China. The range of TCM interventions studied and the assortment of medical diagnoses in which they were assessed were also diverse, with certain most common treatments being observed. The various TCM interventions used and the diseases studied in cohort studies can provide a better reference for clinical practice and point out the direction for further clinical trial implementation and health policy formulation. In many cases, when compared with conventional medicine, the intervention groups were always TCM interventions based on the conventional interventions. When the intervention group were TCM interventions alone, the control groups were mainly other TCM interventions. Future TCM cohort studies should focus more on the weaknesses of conventional medicine, not on replacing it. At the same time, we should fully explore the potential role of TCM therapies in improving patients’ quality of life and symptoms. Twenty-three included TCM cohort studies had involved TCM-related indicators, which was a sound beginning of TCM evaluation.

### Reasons for the low reporting rates

The overall reporting quality of TCM cohort studies was not satisfied, which reflected that the transparency of TCM cohort studies needs to be urgently improved. Comparing with Chinese literature, the reporting rates of English literature in many items were higher. Significantly, there is a lack of reporting of the study size calculation and general statistical methods. Lack of statistical methods description gives the impression the researchers may not familiar with the statistical methods in cohort studies.

The reporting of giving reasons for non-participation and missing data are basic and essential, which can contribute to detecting the potential bias, to judge the accuracy of the results. The low reporting in giving reasons for non-participation and missing data reflected the lack of transparency of data processing.

In observational studies, the quality control of bias should be stricter than other clinical trials [18]. The insufficient reporting reason of the items 18 and 22 are mainly for reporting in the wrong place. According to the STROBE checklist, the summary of results and funding elaboration should be reported in the corresponding place. it’s also related to the lack of recognition of the STROBE statement. The disuse of the flow diagram can be considered for the same reason.

### Inapplicability of the STROBE-cohort checklist

Some statistical methods like subgroup analysis, interaction analysis and sensitivity analysis are seen as essential reporting items according to the STROBE checklist. However, in TCM cohort studies, researchers failed to use these comprehensive methods. As a result, the descriptions of these corresponding analysis were blank. Items 6b, 11 and 16b scored “not applicable” were for the instances. There was an abundance of TCM cohort studies that addressed the effects of the use or not use of TCM interventions but few that focused on the dose gradient of the interventions, which lead to the inapplicability of item 16c. Several items need to take into account the characteristics of TCM interventions. For example, the background and interpretation in the discussion may need the elaboration of TCM rationales; the description of included participants need to describe the TCM diagnosis and syndrome; the detailed information (dose of herbs and formulas, frequency and intensity of acupuncture of the variables) in intervention and control groups should be concerned; the distribution of TCM syndromes may vary with the constitution of the patients, geographically characteristics and seasonal changes which need to be emphasized in generalisability.

### Suggestions

There are several suggestions for improving the reporting quality of TCM cohort studies. 1) The training of statistical methods should be strengthening for the researchers. 2) The protocol and statistical analysis plans should be submitted to the research centers in advance. 3) Researchers, health policymakers, and funders need to enhance their awareness of the STROBE statement. 4) The application of the STROBE statement should be included in the journals related to TCM. 5) Establishing the extension of STROBE-cohort in TCM.

### Limitations

Several limitations to this scoping review need to be noted. We only include the TCM cohort studies published in English and Chinese, exclude some databases like Scopus, which may miss some significant studies published in other languages. We didn’t contact the authors to obtain initial data when we confused by their inconsistency description in the context. Besides, although the process of searching, screening, extracting and evaluating was independently executed by two researchers, the biases may still exist.

## Conclusion

The application of cohort studies to inform the effects of TCM interventions has grown rapidly in recent years, and the diversity of interventions assessed and conditions studied were diverse. The overall reporting quality of TCM cohort studies was poor, the transparency of TCM cohort studies needs to be urgently improved. Future efforts to conduct TCM cohort studies should focus on the statistical methods (including statistical design, control bias, and data transparency) and standardized reporting, as well as the specific characteristics of TCM interventions.

## Supplementary Information

**Additional file 1.** Search strategy for TCM cohort studies.

**Additional file 2.** The overview of basic characteristics of included TCM cohort studies.

**Additional file 3.** Reporting assessment of included TCM cohort studies using the STROBE-cohort checklist.

**Additional file 4.** PRISMA-ScR.

## Data Availability

All data generated or analyzed during this study are included in this published article (and its supplementary information files).

## Abbreviations

TCM: Traditional Chinese Medicine
RCT: Randomized controlled trials
STROBE: Strengthening the Reporting of Observational Studies in Epidemiology
TENS: Transcutaneous electrical nerve stimulation
tVNS: Transcutaneous vagus nerve stimulation
AIDS: Acquired Immune Deficiency Syndrome
EMBASE: Excerpta Medica Database
CENTRAL: Cochrane Central Register of Controlled Trials
AMED: The Allied and Complementary Medicine Database
CBM: Chinese Biomedical Literature Service System
CNKI: China National Knowledge Infrastructure
ICC: Intra-class correlation coefficient
SAR: Special Administration Region
