# Supplementary material for: A scoping review of cohort studies assessing traditional Chinese medicine interventions
Source: BMC Complement Med Ther. 2020 Nov 23;20:361. doi: 10.1186/s12906-020-03150-9 (PMC7684743; doi:10.1186/s12906-020-03150-9)
Supplement: Supplementary file 1 — Additional file 1. Search strategy for TCM cohort studies. [file 12906_2020_3150_MOESM1_ESM.docx]

**Search Strategy for TCM-cohort study**

English databases:

**MEDLINE, EMBASE, CENTRAL, AMED through OVID**

1 (Chinese medicine or herb$).mp.

2 [exp Medicine, Traditional/]

3 ethnobotany.mp. or exp Ethnobotany/

4 phytotherapy.mp. or exp Phytotherapy/

5 Plant Extracts.mp. or exp Plant Extracts/

6 medicinal plant.mp. or exp Plants, Medicinal/

7 Chinese herbal drugs.mp. or exp Drugs, Chinese Herbal/

8 Chinese traditional medicine.mp. or exp Medicine, Chinese Traditional/

9 herbal medicine.mp. or exp Herbal Medicine/

10 (medicinal herb or pharmaceutical plant).mp.

11 (Chinese medicine$ or traditional medicine$).mp.

12 (herbal drug$ or herbal medicine$).mp.

13 (medicinal plant$ or medicinal herb$).mp.

14 (herb$ or herb$ formula$ or decoction$).mp.

15 (herb$ granule$ or herb$ capsule$ or herb$ pellet$).mp.

16 materia medica.mp. or exp Materia Medica/

17 ((single#entity or single) adj3 (component or drug$ or herb$)).mp.

18 (compound prescription$ or herbal mixture or Fufang).mp.

19 (Chinese Medicine Patent Prescription or proprietary Chinese medicines).mp.

20 (Chinese patent adj3 (medicine or drug$)).mp.

21 (Chinese adj3 (patent or proprietary) adj3 (medicine or drug$)).mp.

22 (Chinese adj2 (patent or proprietary) adj2 (medicine or drug$ or prescription$)).mp.

23 or/1-22

24 Acupuncture Therapy/

25 Acupuncture/

26 Pharmacoacupuncture.ti,ab.

27 Electroacupuncture.ti,ab.

28 Electro-acupuncture.ti,ab.

29 Acupuncture Point$.ti,ab.

30 Acupotom$.ti,ab.

31 Needl$.ti,ab.

32 Ear Acupunctures.ti,ab.

33 Auricular Acupuncture.ti,ab.

34 Auricular Acupunctures

35 Moxibustion.mp.

36 Moxibustion therapy

37 Moxa.ti,ab.

38 Cupping.ti,ab.

39 Guasha.ti,ab.

40 Scraping therapy.ti,ab.

41 Massage.mp.

42 Craniosacral Massage.ti,ab.

43 Zone Therap$.ti,ab.

44 Reflexology.ti,ab.

45 Rolfing.ti,ab.

46 Bodywork$.ti,ab.

47 Massage Therap$.ti,ab.

48 Qigong.mp.

49 Qi Gong.ti,ab.

50 Ch'i Kung.ti,ab.

51 Taiji.ti,ab.

52 Baduanjin.ti,ab.

53 Wuqinxi.ti,ab.

54 or/24-53

55 23 or 54

56 Cohort Studies/

57 (Concurrent Studies or Closed Cohort Studies or Cohort Analysis or Historical Cohort Studies or Incidence Studies).ti,ab.

58 56 or 57

59 55 and 58

Chinese databases:

CBM

1. "中草药"[不加权:扩展] OR "复方"[不加权:扩展]) OR "复方合剂"[不加权:扩展]

2. "中药复方"[常用字段] OR ("中药"[常用字段] OR "中草药"[常用字段] OR "中草药提取物"[常用字段] OR "中草药"[主题词])) OR "中医药"[常用字段]) OR "中药组方"[常用字段]) OR ("中草药提取物"[常用字段] OR "中草药"[常用字段] OR "中药"[常用字段] OR "中草药"[主题词])) OR "方"[常用字段]) OR "酒"[常用字段]) OR ("茶"[常用字段] OR "红茶"[常用字段] OR "绿茶"[常用字段] OR "茶"[主题词])) OR "汤"[常用字段]) OR "丸"[常用字段]) OR "散"[常用字段]) OR "颗粒"[常用字段]) OR "

片"[常用字段]) OR ("胶囊"[常用字段] OR "微胶囊"[常用字段] OR "胶囊"[主题词])) OR "经验方"[常用字段]) OR "自拟方"[常用字段]OR”煎”[常用字段] OR “外洗”[常用字段]

3. "针刺"[不加权:扩展])

4. "针刺疗法"[不加权:扩展])

5. "针药"[中文标题:智能]) OR "毫针"[中文标题:智能]

6. "针药"[摘要:智能]) OR "毫针"[摘要:智能]

7. "三棱针"[中文标题:智能]) OR "皮肤针"[中文标题:智能]) OR "梅花针"[中文标题:智能]) OR "皮内针"[中文标题:智能]) OR "火针"[中文标题:智能]) OR "芒针"[中文标题:智能]) OR "鍉针"[中文标题:智能]

8. "三棱针"[摘要:智能]) OR "皮肤针"[摘要:智能]) OR "梅花针"[摘要:智能]) OR "皮内针"[摘要:智能]) OR "火针"[摘要:智能]) OR "芒针"[摘要:智能]) OR "鍉针"[摘要:智能]

9. "耳针"[中文标题:智能]) OR "头针"[中文标题:智能]) OR "眼针"[中文标题:智能]) OR "腹针"[中文标题:智能]) OR "颊针"[中文标题:智能]) OR "腕踝针"[中文标题:智能]) OR "浮针"[中文标题:智能]) OR "平衡针"[中文标题:智能] OR"头皮针"[中文标题:智能]

10. "耳针"[摘要:智能]) OR "头针"[摘要:智能]) OR "眼针"[摘要:智能]) OR "腹针"[摘要:智能]) OR "颊针"[摘要:智能]) OR "腕踝针"[摘要:智能]) OR "浮针"[摘要:智能]) OR "平衡针"[摘要:智能]OR"头皮针"[中文标题:智能]

11. "电针"[中文标题:智能]) OR "激光针"[中文标题:智能]) OR "微波针"[中文标题:智能]) OR "经皮穴位电刺激"[中文标题:智能]) OR "五行针"[中文标题:智能]) OR "蜂针"[中文标题:智能]

12. "电针"[摘要:智能]) OR "激光针"[摘要:智能]) OR "微波针"[摘要:智能]) OR "经皮穴位电刺激"[摘要:智能]) OR "五行针"[摘要:智能]) OR "蜂针"[摘要:智能]

13. "穴位埋线"[中文标题:智能]) OR "穴位注射"[中文标题:智能]

14. "穴位埋线"[摘要:智能]) OR "穴位注射"[摘要:智能]

15. "灸法"[不加权:扩展])

16. "灸法"[常用字段:智能] OR "艾灸"[常用字段:智能] OR "艾柱"[常用字段:智能] OR "艾条"[常用字段:智能] OR "艾疗"[常用字段:智能] OR "艾灸疗法"[常用字段:智能] OR "灸疗"[常用字段:智能]OR"隔物灸"[常用字段:智能] OR "隔姜灸"[常用字段:智能] OR "隔蒜灸"[常用字段:智能] OR "热敏灸"[常用字段:智能] OR "精灸"[常用字段:智能] OR "麦粒灸"[常用字段:智能] OR "隔附子饼灸"[常用字段:智能]

17. "拔罐" [不加权:扩展]

18. "拔罐"[常用字段:智能] OR "拔罐疗法"[常用字段:智能] OR "罐疗"[常用字段:智能] OR "湿罐"[常用字段:智能] OR "药罐"[常用字段:智能]OR "走罐"[常用字段:智能] OR "针罐"[常用字段:智能] OR"刺络"[常用字段:智能] OR "刺络放血"[常用字段:智能] OR "放血"[常用字段:智能]

19. "刮痧"[常用字段:智能] OR "刮法"[常用字段:智能] OR "刮痧疗法"[常用字段:智能]

OR "痧疗"[常用字段:智能] OR "刮痧治法"[常用字段:智能]

20. "推拿"[常用字段:智能] OR "按摩"[常用字段:智能] OR "整脊"[常用字段:智能] OR "正骨"[常用字段:智能] OR "接骨"[常用字段:智能] OR "案杌"[常用字段:智能] OR "按跷"[常用字段:智能]

21. "气功"[常用字段:智能] OR "太极"[常用字段:智能] OR "太极拳"[常用字段:智能] OR "五禽戏"[常用字段:智能] OR "易筋经"[常用字段:智能] OR "八段锦"[常用字段:智能]

22. =or/1-21

23. "队列研究"[不加权:扩展]

24. ((((("定群研究"[常用字段:智能]) OR "定群分析"[常用字段:智能]) OR "同时性研究"[常用字段:智能]) OR "发病率研究"[常用字段:智能]) OR "随访研究"[常用字段:智能]) OR "纵向研究"[常用字段:智能]

25. =23or24

26. =22and25

VIP

((U = “复方” OR U = “复方合剂” OR U=“中草药” OR M = 中药复方 + 中药 +中药组方 + 方 + 经方 + 验方 + 经验方 + 汤 + 自拟方 + 中成药 + 颗粒剂 + 丸 + 散 + 中医药 + 中草药提取物 OR R = 中药复方 + 中药 + 中药组方 + 方 + 经方 + 验方 + 经验方 + 汤 + 自拟方 + 中成药 + 颗粒剂 + 丸 +散 + 中医药 + 中草药提取物+ 煎 + 外洗) OR (U = 针刺OR U = 针刺疗法 OR M = 针药 + 毫针 + 三棱针 + 皮肤针 + 梅花针 + 皮内针 + 火针 + 芒针 + 鍉针 + 耳针 + 头针 + 眼针 + 腹针 + 颊针 + 腕踝针 + 浮针 + 平衡针 + 电针 + 激光针 + 微波针 + 经皮穴位电刺激 + 五行针 + 蜂针 + 穴位埋线 + 穴位注射 OR R = 针药 + 毫针 + 三棱针 + 皮肤针 + 梅花针 + 皮内针 + 火针 + 芒针 + 鍉针 + 耳针 + 头针 + 眼针 + 腹针 + 颊针 + 腕踝针 + 浮针 + 平衡针 + 电针 + 激光针 + 微波针 + 经皮穴位电刺激 + 五行针 + 蜂针 + 穴位埋线 + 穴位注射) OR (U = “灸法” OR U = 艾灸疗法 OR U = “灸疗” OR M = 艾柱 +艾条 + 针灸 + 针灸结合 + 直接灸 + 间接灸 + 隔物灸 +隔姜灸 + 隔蒜灸 + 热敏灸 + 精灸 + 麦粒灸 + 隔附子饼灸) OR (U = “拔罐” OR U = 拔罐疗法 OR M = 罐疗 +湿罐 + 药罐 + 针罐 + 走罐 + 刺络 + 刺络放血 + 放血) OR (M = 刮痧 + 痧疗 + 刮痧疗法) OR (M = 推拿 + 按摩 + 整脊 + 正骨 + 接骨 + 案杌 + 按跷) OR (M = 气功 + 八段锦 + 五禽戏 + 易筋经 + 太极拳 + 太极) AND ( U = 队列研究 OR M = 定群研究 + 定群分析 + 同时性研究 + 发病率研究 + 随访研究 OR R = 纵向研究)

万方

(主题:("复方")+主题:("复方合剂")+题名或关键词:(中药复方)+题名或关键词:(中药)+题名或关键词:(中药组方)+题名或关键词:(方)+题名或关键词:(经方)+题名或关键词:(验方)+题名或关键词:(经验方)+题名或关键词:(汤)+题名或关键词:(自拟方)+题名或关键

词:(中成药)+题名或关键词:(颗粒剂)+题名或关键词:(丸)+题名或关键词:(散)+题名或关键词:(中医药)+题名或关键词:(中草药)+题名或关键词:(中草药提取物) + 题名或关键词:(煎)+题名或关键词:(外洗) +主题:("针刺") + 主题:("针刺疗法") + 题名或关键词:(针药) + 题名或关键词:(毫针) + 题名或关键词:(三棱针) + 题名或关键词:(皮肤针) + 题名或关键词:(梅花针) + 题名或关键词:(皮内针) + 题名或关键词:(火针) + 题名或关键词:(芒针) + 题名或关键词:(鍉针) + 题名或关键词:(耳针) + 题名或关键词:(头针) + 题名或关键词:(眼针) + 题名或关键词:(腹针) + 题名或关键词:(颊针) + 题名或关键词:(腕踝针) + 题名或关键词:(浮针) + 题名或关键词:(平衡针) + 题名或关键词:(电针) + 题名或关键词:(激光针) + 题名或关键词:(微波针) + 题名或关键词:(经皮穴位电刺激) + 题名或关键词:(五行针) + 题名或关键词:(蜂针) + 题名或关键词:(穴位埋线) + 题名或关键词:(穴位注射))+ (主题:("灸法") + 题名或关键词:(针灸) + 题名或关键词:(针灸疗法) + 题名或关键词:(灸疗) + 题名或关键词:(艾疗) + 题名或关键词:(艾灸疗法) + 题名或关键词:(艾柱) + 题名或关键词:(艾条) + 题名或关键词:(直接灸) + 题名或关键词:(间接灸) + 主题:("拔罐") + 题名或关键词:(罐疗) + 题名或关键词:(湿罐) + 题名或关键词:(药罐) + 题名或关键词:(拔罐疗法) + 题名或关键词:(走罐) + 题名或关键词:(针罐) + 题名或关键词:(刺络) + 题名或关键词:(刺络放血) + 题名或关键词:(放血) + 题名或关键词:(刮痧) + 题名或关键词:(刮痧疗法) + 题名或关键词:(刮法) + 题名或关键词:(痧疗) + 题名或关键词:(刮痧治法) + 主题:("推拿") + 题名或关键词:(整脊) + 题名或关键词:(按摩) + 题名或关键词:(正骨) + 题名或关键词:(接骨) + 题名或关键词:(案杌) + 题名或关键词:(按跷) + 题名或关键词:(气功) + 题名或关键词:(功法) + 题名或关键词:(五禽戏) + 题名或关键词:(八段锦) + 题名或关键词:(易筋经) + 题名或关键词:(太极) + 题名或关键词:(太极拳))*(主题:("队列研究")+题名或关键词:(定群研究)+题名或关键词:(定群分析)+题名或关键词:(同时性研究)+题名或关键词:(发病率研究)+题名或关键词:(随访研究)+题名或关键词:(纵向研究))

CNKI

(SU = 中草药+复方+复方合剂+针刺+灸法+拔罐+刮痧+气功 OR TI= 中草药+中药复方+复方+复方合剂+中药+中医药+中药组方+中草药提取物+方+酒+茶+汤+丸+散+颗粒+片+胶囊+经验方+自拟方+煎+外洗+针药 + 毫针 + 三棱针 + 皮肤针 + 梅花针 + 皮内针 + 火针 + 芒针 + 鍉针 + 耳针 + 头针 + 眼针 + 腹针 + 颊针 + 腕踝针 + 浮针 + 平衡针 + 电针 + 激光针 + 微波针 + 经皮穴位电刺激 + 五行针 + 蜂针 + 穴位埋线 + 穴位注射 OR R = 针药 + 毫针 + 三棱针 + 皮肤针 + 梅花针 + 皮内针 + 火针 + 芒针 + 鍉针 + 耳针 + 头针 + 眼针 + 腹针 + 颊针 + 腕踝针 + 浮针 + 平衡针 + 电针 + 激光针 + 微波针 + 经皮穴位电刺激 + 五行针 + 蜂针 + 穴位埋线 + 穴位注射 + 艾灸疗法 + 艾疗 + 灸疗 + 温针灸 + 药灸 + 直接灸 + 间接灸 + 刮痧 + 痧疗 + 刮痧疗法 + 罐疗 + 湿罐 + 药罐 + 针罐 + 走罐 +拔罐疗法 + 放血 + 刺络 + 刺络放血+ 推拿 + 按摩 + 整脊 + 正骨 + 接骨 + 案杌 + 按跷 + 太极 + 气功 + 五禽戏 + 八段锦 + 易筋经 OR KY =中草药+中药复方+复方+复方合剂+中药+中医药+中药组方+中草药提取物+方+酒+茶+汤+丸+散+颗粒+片+胶囊+经验方+自拟方+煎+外洗+针药 + 毫针 + 三棱针 + 皮肤针 + 梅花针 + 皮内针 + 火针 + 芒针 + 鍉针 + 耳针 + 头针 + 眼针 + 腹针 + 颊针 + 腕踝针 + 浮针 + 平衡针 + 电针 + 激光针 + 微波针 + 经皮穴位电刺激 + 五行针 + 蜂针 + 穴位埋线 + 穴位注射 OR R = 针药 + 毫针 + 三棱针 + 皮肤针 + 梅花针 + 皮内针 + 火针 + 芒针 + 鍉针 + 耳针 + 头针 + 眼针 + 腹针 + 颊针 + 腕踝针 + 浮针 + 平衡针 + 电针 + 激光针 + 微波针 + 经皮穴位电刺激 + 五行针 + 蜂针 + 穴位埋线 + 穴位注射 + 艾灸疗法 + 艾疗 + 灸疗 + 温针灸 + 药灸 + 直接灸 + 间接灸 + 刮痧 + 痧疗 + 刮痧疗法 + 罐疗 + 湿罐 + 药罐 + 针罐 + 走罐 +拔罐疗法 + 放血 + 刺络 + 刺络放血+ 推拿 + 按摩 + 整脊 + 正骨 + 接骨 + 案杌 + 按跷 + 太极 + 气功 + 五禽戏 + 八段锦 + 易筋经) AND (SU = 队列研究 OR TI= 队列研究+定群研究+同时性研究+发病率研究+随访研究+纵向研究 OR KY=队列研究+定群研究+同时性研究+发病率研究+随访研究+纵向研究)
