# Supplementary material for: A scoping review of cohort studies assessing traditional Chinese medicine interventions
Source: BMC Complement Med Ther. 2020 Nov 23;20:361. doi: 10.1186/s12906-020-03150-9 (PMC7684743; doi:10.1186/s12906-020-03150-9)
Supplement: Supplementary file 2 — Additional file 2. The overview of basic characteristics of included TCM cohort studies. [file 12906_2020_3150_MOESM2_ESM.doc]

| Publication year | Author | Country/Regions | Journal | Clinical conditions | Research purposes | Study design | Intervention group | Control group | Outcomes | | Study size | | Database | Funding | Publication language |
| --- | --- | --- | --- | --- | --- | --- | --- | --- | --- | --- | --- | --- | --- | --- | --- |
|  |  |  |  |  |  |  |  |  | General outcomes | Traditional Chinese Medicine outcomes | Intervention group | Control group |  |  |  |
| 2019 | ZHOU Yuchen[1] | Mainland China | J South Med Univ | Infectious diseases(chronic hepatitis B) | Prognosis | Prospective cohort study | Chinese herbal medicine formulas( Biejiaruangan tablet)* | Positive control | HBV DNA levels/liver stiffness measurement | no | Adefovir with Biejiaruangan tablet group: 60; entecavir with Biejiaruangan tablet group: 180; tenofovir with Biejiaruangan tablet group: 135 | Adefovir group: 58; entecavir group: 185; tenofovir group: 131 | no | yes | Chinese |
| 2019 | FANG Yanyan[2] | Mainland China | Chinese Journal of Integrated Traditional and Western Medicine | Immune system diseases(ankylosing spondylitis) | Effect evaluation | Retrospective cohort study | Chinese herbal medicine formulas(Unspecified)* | Positive control | Endpoint events | no | High Exposure Group: 159; Mid Exposure Group: 38; Low Exposure Group:70 | 56 | no | yes | Chinese |
| 2019 | LI Lei[3] | Mainland China | World Journal of Integrated Traditional and Western Medicine | Nervous system diseases(insomnia) | Effect evaluation | Prospective cohort study | Chinese herbal medicine formulas(nourishing kidney, harmonizing liver and spleen and tranquilizing mind ) | Blank control | Total effective rate/Pittsburgh Sleep Quality Index( PSQI) factor | TCM symptoms score | 38 | 14 | no | yes | Chinese |
| 2019 | MENG ShuHui[4] | Mainland China | CHINA JOURNAL OF PHARMACEUTICAL ECONOMICS | Nervous system diseases(Stroke) | Economic evaluation | Prospective cohort study | Chinese herbal medicine formulas (Getong Tongluo Capsule) | Other Traditional Chinese Medicine intervention(Yinxingye Dripping Pills) | Rankin revised scale score (mRS)/Activity of daily living ability (Barthel index)/National Institute of Health stroke scale (NIHSS)/Quality adjusted life years (QALYs) | no | 288 | 152 | no | yes | Chinese |
| 2019 | LIU Sutong[5] | Mainland China | ACTA CHINESE MEDICINE | Oncology diseases(Lung Cancer) | Prognosis | Retrospective cohort study | Chinese herbal medicine formulas(Fuzheng Quxie Prescription)* | Positive control | Progression-free survival | no | 360 | 351 | no | yes | Chinese |
| 2019 | ZHANG Liang[6] | Mainland China | Journal of Traditional Chinese Medicine | Oncology diseases(hepatocellular carcinoma) | Prognosis | Retrospective cohort study | Chinese herbal medicine formulas(Huqi formulas)* | Positive control | Quality of Life Score/Laboratory indicators of liver | TCM symptoms score | 88 | 89 | no | yes | Chinese |
| 2019 | Miao Ruiheng[7] | Mainland China | Chinese Journal of health medicine. | Motor system diseases(Myofascial pain syndrome) | Effect evaluation | Retrospective cohort study | Acupuncture | Other TCM intervention | Life quality：NRS(Numeric rating scales ) | no | 27 | 67 | no | yes | Chinese |
| 2019 | Jiao Huiduo[8] | Mainland China | World Chinese Medicine. | Nervous system diseases(Stroke) | Effect evaluation | Retrospective cohort study | Acupuncture* | Positive control | 1.Bioindicator:①NSE(Neuron-specific enolas);②H2S;③NGF(Nerve growth factor);④CVHD(Cerebral vascular hemodynamic)；2.Life quality:①FMA(Fugl-Meyer score);② MBI(Modified barthel index) | TCM symptoms score | 100 | 98 | no | yes | Chinese |
| 2019 | Che-Jui Chang[9] | Taiwan | environmental reseacher and public health | Digestive system diseases(Stomach cancer) | Adverse events | Retrospective cohort study | Single herbs (Talc Powder without Asbestos via) | Blank control | Diagnosed with stomach cancer (the event) | no | 21575 | 584077 | National Health Insurance Research Database | yes | English |
| 2019 | Tao Shen[10] | mainland China | Gastroenterology | Digestive system diseases(Drug-induced liver injury) | Adverse events | Retrospective cohort study | Two and above TCM interventions | No control | No access to get data | no | No access to get data | No access to get data | no | yes | English |
| 2019 | GONG Zhao-hui[11] | Mainland China | Journal of Traditional Chinese Medicine | Circulatory system diseases(acute myocardial infarction) | Effect evaluation | Retrospective cohort study | Chinese herbal medicine formulas (Tongmai Buxin Ointment)* | Positive control | Killip grading，New York Heart Association (NYHA) cardiac function grading，serum B-type brain natri-uretic peptide (BNP) level，cardiac color Doppler ultrasound results including left ventricular ejection fraction(LVEF)，left ventricular end diastolic diameter (LVEDD)，left ventricular end systolic diameter (LVESD)，and quality of life evaluation before and after treatment | TCM symptoms score | 50 | 49 | no | yes | Chinese |
| 2019 | CUI Wei-feng[12] | Mainland China | Chinese General Practice | Circulatory system diseases(hypertension) | Effect evaluation | Prospective cohort study | Chinese herbal medicine formulas (Jiangyabao Series) (long-term administration >42 months) | TCM intervention (dose control)（Jiangyabao Series）(short-term administration ≤ 42 months) | the incidence of adverse outcomes （death caused by cardiovascular and cerebrovascular diseases，myocardial infarction，cerebral infarction，cerebral hemorrhage） | no | 1128 | 236 | no | yes | Chinese |
| 2019 | Zhang Li-jie[13] | Mainland China | J Hunan Normal Univ(Med Sci) | Digestive system diseases(compensatory period of hepatitis b cirrhosis) | Effect evaluation | Prospective cohort study | Chinese herbal medicine formulas(unspecified)(medication≥8month every year+2≤medication<8month every year) | Blank control(including<2month every year) | Knodell score, APRI score, Child score, B-ultrasound score;The generalized reversal rate of patients with compensatory phase of hepatitis B liver cirrhosis | TCM symptoms score | 77 | 22 | no | yes | Chinese |
| 2019 | Liang-Yu Chen[14] | Taiwan | Journal of Affective Disorders | Others(depression) | Prognosis | Retrospective cohort study | Acupuncture | Blank control | stroke incidence | no | 13823 | 13823 | National Health Insurance Research Database | yes | English |
| 2018 | Ching-Hui Huang[15] | Taiwan | Journal of Ethnopharmacology | Oncology diseases(breast cancer) | Prognosis | Retrospective cohort study | Chinese herbal medicine formulas(Unspecified)* | Positive control | Cumulative incidence of congestive heart failure(CHF) | no | 24457 | 24457 | National Health Insurance Research Database | yes | English |
| 2018 | Su-Tso Yang[16] | Taiwan | BMC Complementary and Alternative Medicine | Infectious diseases(tuberculosis) | Effect evaluation | Retrospective cohort study | Chinese herbal medicine formulas(Unspecified)* | Positive control | Prescription of Chinese medicines | no | 2051 | 1874 | National Health Insurance Research Database | yes | English |
| 2018 | WEN Jianting[17] | Mainland China | World Journal of Integrated Traditional and Western Medicine | Immune system diseases(ankylosing spondylitis) | Prognosis | Retrospective cohort study | Chinese herbal medicine formulas(Unspecified)* | Positive control | Endpoint events/Laboratory indicators | no | High Exposure Group: 159; Mid Exposure Group: 38; Low Exposure Group:70 | 56 | no | yes | Chinese |
| 2018 | FANG Yanyan[18] | Mainland China | Chin J Clin Healthc | Immune system diseases(Rheumatoid Arthritis) | Effect evaluation | Prospective cohort study | Chinese herbal medicine formulas(Unspecified)* | Positive control | Endpoint events | no | High Exposure Group: 943; Mid Exposure Group: 187; Low Exposure Group:162 | 176 | no | yes | Chinese |
| 2018 | XU Zhigang[19] | Mainland China | InnerMongolia Journal of Traditional ChineseMedicine | Immune system diseases(Rheumatoid Arthritis) | Effect evaluation | Retrospective cohort study | Chinese herbal medicine formulas(Shaogua Xiegong Duan cao Decoction)* | Positive control | Efficiency rate | Evaluation of TCM Syndrome Integral | 30 | 30 | no | yes | Chinese |
| 2018 | CHEN Menglian[20] | Mainland China | Herald of Medicine | Circulatory system diseases(primary hypertension) | Effect evaluation | Prospective cohort study | Two and above TCM interventions * | Positive control | Hypertension control rate | no | 98 | 100 | no | yes | Chinese |
| 2018 | YUAN Chenhao[21] | Mainland China | JOURNAL OF SHANDONG UNIVERSITY OF TCM | Circulatory system diseases(Myocardial Infarction) | Prognosis | Retrospective cohort study | Chinese herbal medicine formulas(Compound Danshen Dripping Pills)* | Positive control | Endpoint events | no | High Exposure Group: 72; Mid Exposure Group: 64; Low Exposure Group:71 | 1003 | no | yes | Chinese |
| 2018 | Wen Jianting[22] | Mainland China | Chin J Clin Healthc | Motor system diseases(Osteoarthritis) | Prognosis | Retrospective cohort study | Chinese herbal medicine formulas(Unspecified)* | Positive control | Endpoint events | no | High Exposure Group: 822; Mid Exposure Group: 292; Low Exposure Group:204 | 340 | no | yes | Chinese |
| 2018 | Lin Ruizhu[23] | Mainland China | West China Medical Journal. | Nervous system diseases(Neurogenic bladder after spinal cord injury) | Effect evaluation | Retrospective cohort study | Moxibustion* | Positive control | 1.Bioindicator: Routine urine leucocyte count；2.Life quality：①Urination diary related indicators；②ICI-Q-SF(International urinary incontinence advisory committee urinary incontinence questionnaire short form) | no | Group(Double moxibustion combined with intermittent catheterization)86 | 70 | no | yes | Chinese |
| 2018 | LUO Xiaozhou[24] | Mainland China | Journal of Basic Chinese Medicine. | Motor system diseases(Periarthritis of Shoulder) | Effect evaluation | Prospective cohort study | Acupuncture | Other TCM intervention | 1.Efficacy；2.Life quality：① Michael feeds shoulder joint function score；②VAS(Visual analogue score)；③ADL(Activities of daily living) | no | 39 | 30 | no | yes | Chinese |
| 2018 | ZHU Dao-cheng[25] | Mainland China | Acupuncture Research. | Nervous system diseases(Facial Paralysis) | Effect evaluation | Prospective cohort study | Two and above TCM interventions | Other TCM intervention | Life quality:Portmann scores | no | 33 | 33 | no | yes | Chinese |
| 2018 | ZHANG Ye[26] | Mainland China | Journal of Basic Chinese Medicine. | Others(Depression) | Effect evaluation | Prospective cohort study | Acupuncture | Positive control | Life quality:①HAMD(The hamilton depression rating scale);②SF-36(The Medical Outcomes Study Short Form) | no | 35 | 25 | no | yes | Chinese |
| 2018 | Zhixin Y[27] | Mainland China | Journal of External Therapy of TCM. | Nervous system diseases(Peripheral nerve injury after chemotherapy) | Effect evaluation | Prospective cohort study | Two and above TCM interventions | Positive control | 1.Efficacy rates；2.Syndrome score | no | 28 | 27 | no | no | Chinese |
| 2018 | Zhang Wei[28] | Mainland China | Journal of Clinical Acupuncture and Moxibustion. | Nervous system diseases(Ischemic Stroke) | Effect evaluation | Prospective cohort study | Acupuncture* | Positive control | Life quality：①FMA-UE(The upper-extremity portion of the fugl meyer motor assessment,Fugl-Meyer);②FTHUE-HK(Functional test for the hemiplegic upper extremity-Hong Kong);③MBI(Modified barthel index) | no | 56 | 50 | no | no | Chinese |
| 2018 | GAO Wu-lin[29] | Mainland China | Chinese Journal of Experimental Traditional Medical Formulae. | Circulatory system diseases(Unstable Angina) | Effect evaluation | Prospective cohort study | Chinese herbal medicine formulas(unspecified) | Positive control | End-point event rate | no | 773 | 232 | no | yes | Chinese |
| 2018 | GAO Wu-lin[30] | Mainland China | Li Shi Zhen Medicine and Materia Medica Research. | Circulatory system diseases(Myocardial Infarction Complicated with Hypertension) | Effect evaluation | Prospective cohort study | Chinese herbal medicine formulas(unspecified)* | Positive control | End-point event rate | no | 1290 | 423 | no | yes | Chinese |
| 2018 | GAO Wu-lin[31] | Mainland China | China Journal of Traditional Chinese Medicine and Pharmacy. | Circulatory system diseases(Combination disease of myocardial infarction and hyperlipidemia) | Effect evaluation | Prospective cohort study | Chinese herbal medicine formulas(unspecified)* | Positive control | End-point event rate | no | 956 | 289 | no | yes | Chinese |
| 2018 | YUAN Jun[32] | Mainland China | Chinese Journal of Integrated Traditional and Western Medicine. | Immune system diseases(AIDS) | Effect evaluation | Retrospective cohort study | Chinese herbal medicine formulas(Yiaikang capsule)* | Positive control | Bioindicator:CD4+ lymphocyte count | no | 323 | 164 | no | yes | Chinese |
| 2018 | CHEN Yang[33] | Mainland China | Journal of Hepatobiliary Surgery. | Oncology diseases(Hepatocellular carcinoma) | Prognosis | Retrospective cohort study | Chinese herbal medicine formulas(unspecified)* | Positive control | Survival related indicators:Overall survival | no | Group1(High exposure):34;Group2:(Exposure):56;Group3(Low exposure):35 | 32 | no | no | Chinese |
| 2018 | Irmgard Simma[34] | Austria | Acupunct Med | Motor system diseases(Temporomandibular disorders) | Effect evaluation | Retrospective cohort study | Acupuncture | No control | Life quality:Likert scale (No pain to strong pain) | no | 407 | no | no | no | English |
| 2018 | DONG Wen-zhe[35] | Mainland China | Rheumatism and Arthritis | Immune system diseases(rheumatoid arthritis) | Effect evaluation | Retrospective cohort study | Chinese herbal medicine formulas(unspecified) | Blank control | end-point events | no | 1292 | 176 | no | yes | Chinese |
| 2018 | Li Xin[36] | Mainland China | Chin J Prev Med | Oncology diseases(lung cancer) | Effect evaluation | Prospective cohort study | Single herbs(Tea) | Blank control | lung cancer risk | no | 43084 | 59926 | no | yes | Chinese |
| 2018 | ZHENG Shi-jing[37] | Mainland China | WORLD CHINESE MEDICINE | Urinary system diseases(chronic nephritis) | Effect evaluation | Prospective cohort study | Chinese herbal medicine formulas(Renal collateral remove lump) | Other TCM intervention(Conventional Syndrome Differentiation) | the glomerular filtration rate and increase rate;the creatinine level;urea nitrogen;the 24h urine protein | TCM syndrome score | 208 | 102 | no | yes | Chinese |
| 2018 | TIAN Zhong-hua[38] | Mainland China | LISHIZHEN MEDICINE AND MATERIA MEDICA RESAERCH | Nervous system diseases(recurrent stroke) | Effect evaluation | Prospective cohort study | Chinese herbal medicine formulas( ZhongFeng Capsule)(Take more than 24 months) | TCM intervention (dose control)( ZhongFeng Capsule)(Take less 24 months) | outcome events(cerebral infarction，cerebral hemorrhage，death) | no | 295 | 321 | no | yes | Chinese |
| 2018 | DENG Peng[39] | Mainland China | Journal of Traditional Chinese Medicine | Circulatory system diseases(chronic stable angina) | Effect evaluation | Retrospective cohort study | Chinese herbal medicine formulas(Medicinal liquor)* | Positive control | the attack time and duration of angina pectoris，electrocardiogram outcome | TCM syndrome score | 50 | 49 | no | yes | Chinese |
| 2018 | Mei-Yao Wu[40] | Taiwan | BMC Complementary and Alternative Medicine | Immune system diseases(rheumatoid arthritis) | Prognosis | Retrospective cohort study | Acupuncture | Blank control | incidence of CHD | no | 9932 | 9932 | National Health Insurance Research Database | yes | English |
| 2017 | Yi-Ting Kuo[41] | Taiwan | Integrative Cancer Therapies | Oncology diseases(Pancreatic Cancer) | Prognosis | Retrospective cohort study | Chinese herbal medicine formulas(Unspecified)* | Positive control | Mortality rate/Survival rate | no | 386 | 386 | Taiwanese Registry for Catastrophic Illness Patients Database | yes | English |
| 2017 | AIHEMAITI.Abudureyimu[42] | Mainland China | Chinese Journal of Medicinal Guide | Infectious diseases(AIDS) | Effect evaluation | Prospective cohort study | Chinese herbal medicine formulas(Taiqipeiyuan Granules)* | Positive control | CD4+ T lymphocytes/HIV-RNA viral load/TLR-4 receptor | TCM symptoms score | 54 | 54 | no | yes | Chinese |
| 2017 | WEI Yeye[43] | Mainland China | Chinese Journal of Medicinal Guide | Infectious diseases(AIDS) | Effect evaluation | Prospective cohort study | Chinese herbal medicine formulas(Taiqipeiyuan Granules)* | Positive control | CD4+ T lymphocytes/HIV-RNA viral load/TLR-4 receptor | TCM symptoms score | 81 | 10 | no | yes | Chinese |
| 2017 | MENG Yuan[44] | Mainland China | The Journal of Practical Medicine | Urinary system diseases(chronic kidney disease) | Effect evaluation | Retrospective cohort study | Chinese herbal medicine formulas(qingbudigui decoction)* | Positive control | SCr/eGFR/U⁃Pro | no | 52 | 51 | no | yes | Chinese |
| 2017 | WEN Jianting [45] | Mainland China | J ANHUI UNIV CHINESE MED | Immune system diseases(Rheumatoid Arthritis) | Prognosis | Retrospective cohort study | Chinese herbal medicine formulas(Unspecified)* | Positive control | Endpoint events | no | High Exposure Group: 943; Mid Exposure Group: 187; Low Exposure Group:162 | 176 | no | yes | Chinese |
| 2017 | ZHU Haitao[46] | Mainland China | JOURNAL OF GUIZHOU MEDICAL UNIVERSITY | Digestive system diseases(Mild Acute Pancreatitis) | Effect evaluation | Prospective cohort study | Single herbs(Rhubarb)* | Positive control | Remission time of symptom and Laboratory indicators | no | biliary mild AP combined treatment group: 17; hyperlipidemia AP combined treatment group: 14; idiopathic mild AP combined treatment group: 7 | biliary mild AP standard treatment group: 15; hyperlipidemia AP standard treatment group: 14; idiopathic mild AP standard treatment group:12 | no | yes | Chinese |
| 2017 | JIANG Fangchao[47] | Mainland China | Jilin Journal of Chinese Medicine | Circulatory system diseases(hypertension) | Effect evaluation | Prospective cohort study | Chinese herbal medicine formulas(Bushen Jiangzhuo decoction)* | Positive control | Hypertension control rate /montreal cognitiveassessment | TCM symptoms score | 158 | 97 | no | yes | Chinese |
| 2017 | FANG Yanyan[48] | Mainland China | Rheumatism and Arthritis | Motor system diseases(Osteoarthritis) | Effect evaluation | Retrospective cohort study | Chinese herbal medicine formulas(Formula of Fortifying the Spleen and Removing Dampness,Tonifying Kidney and Dredging Collaterals)* | Positive control | Endpoint events | no | High Exposure Group: 822; Mid Exposure Group: 292; Low Exposure Group:204 | 340 | no | yes | Chinese |
| 2017 | WANG Haibing[49] | Mainland China | Chinese Journal of Drug Evaluation | Motor system diseases(Soft Blood Tissue Injury) | Economic evaluation | Prospective cohort study | Chinese herbal medicine formulas(Huoxue Zhitong Soft Capsule) | Other Traditional Chinese Medicine intervention(Unspecified) | Rate of pain loss/Cost | no | 302 | 297 | no | no | Chinese |
| 2017 | TAO Li[50] | Mainland China | Shanghai Journal of Traditional Chinese Medicine | Oncology diseases(Colorectal cancer) | Effect evaluation | Prospective-Retrospective cohort study | Chinese herbal medicine formulas(Weichang’an Prescription)* | Positive control | Overall survival/Progression-free survival | no | 65 | 66 | no | yes | Chinese |
| 2017 | Haiyan Xu[51] | Mainland China | Liaoning Journal of Traditional Chinese Medicine. | Motor system diseases(Periarthritis of Shoulder) | Effect evaluation | Prospective cohort study | Moxibustion | Other TCM intervention | 1.Efficacy;2.Life quality：① Michael feeds shoulder joint function score；②MPQ(McGill pain questionaire,McGill)；③ADL(Activities of daily living) | no | 30 | 30 | no | yes | Chinese |
| 2017 | Xu Wei[52] | Mainland China | Jiang Xi Journal of Traditional Chinese Medicine. | Motor system diseases(Knee osteoarthritis) | Effect evaluation | Prospective cohort study | Moxibustion | Other TCM intervention | Life quality:①VAS(Visual analogue score);②Lysholm knee score(Lysholm) | no | 34 | 32 | no | yes | Chinese |
| 2017 | Wang Li[53] | Mainland China | Journal of Anhui University of Chinese Medicine. | Pediatric diseases(Asthma) | Effect evaluation | Prospective cohort study | Acupoint application | Blank control | Syndrome score | no | 196 | 133 | no | yes | Chinese |
| 2017 | Wang Jianbin[54] | Mainland China | Journal of Medical Research. | Oncology diseases(Primary Liver Cancer) | Prognosis | Retrospective cohort study | Chinese herbal medicine formulas(unspecified)* | Positive control | Survival related indicators:①Relapse rates;②Metastasis rates;③Survival rates | no | Group1(High exposure):105;Group2(Low exposure):93 | 107(non-exposure) | no | yes | Chinese |
| 2017 | XUE Mei－ping[55] | Mainland China | World Journal of Integrated Traditional and Western Medicine. | Oncology diseases(Small cell lung cancer) | Effect evaluation | Prospective cohort study | Chinese herbal medicine formulas(unspecified) | Blank control | 1.Efficacy;2.Survival related indicators:①PFS(Progression-free-survival span);②KPS(Karnofsky performance score) | no | 30 | 30 | no | no | Chinese |
| 2017 | Yin-Yin Lin[56] | Taiwan | Journal of Ethnopharmacology | Oncology diseases(Colon cancer) | Effect evaluation | Retrospective cohort study | Chinese herbal medicine formulas(Finished herbal products (FHP) of danshen） | TCM intervention (dose control：less or more than 84 grams) | Survival related indicators:Survival rate | no | 1366 | 55599 | National Health Insurance Research Database | no | English |
| 2017 | Tsai-Hui Lin[57] | Taiwan | Journal of Ethnopharmacology | Oncology diseases(Chronic hepatitis in colon cancer) | Effect evaluation | Retrospective cohort study | Chinese herbal medicine formulas(unspecified)* | Positive control | Incidence and cumulative incidence of chronic hepatitis | no | 1624 | 1624 | National Health Insurance Research Database | yes | English |
| 2017 | Qi Shi[58] | Mainland China | Oncotarget | Oncology diseases(Postoperative stage I-III colorectal patients) | Effect evaluation | Retrospective cohort study | Chinese herbal medicine formulas(unspecified)* | Positive control | Survival related indicators:Survival rate | no | 523 | 294 | no | no | English |
| 2017 | Kuo-Chin Huang[59] | Taiwan | Evidence-Based Complementary and Alternative Medicine | Oncology diseases(Chronic hepatitis in patients with breast cancer) | Effect evaluation | Retrospective cohort study | Two and above TCM interventions * | Positive control | Incidence of chronic hepatitis | no | 8918 | 12152 | National Health Insurance Research Database | yes | English |
| 2017 | Jiang Zhi-yan[60] | Mainland China | WORLD CHINESE MEDICINE | Respiratory system diseases(Pediatric Mycoplasma Pneumonia) | Effect evaluation | Prospective cohort study | Chinese herbal medicine formulas(unspecified)* | Positive control | no | TCM symptoms score | 310 | 90 | no | yes | Chinese |
| 2017 | WANG Pei-yu[61] | Mainland China | Journal of Wenzhou Medical University | Gynecological diseases(ovarian hyper-stimulation syndrome) | Effect evaluation | Prospective cohort study | Chinese herbal medicine formulas（Wuling powder combined with Wupi drink）** | Positive control | laboratory and clinical outcomes, vascular endothelial growth factor(VEGF), interleukin-6 (IL-6) and Nitric oxide(NO) | no | 50 | 24 | no | yes | Chinese |
| 2017 | DONG Ya-nan[62] | Mainland China | Journal of Yunnan University of Traditional Chinese Medicine | Digestive system diseases(decompensation of hepatitis bcirrhosis) | Effect evaluation | Retrospective cohort study | Chinese herbal medicine formulas(unspecified)* | Positive control | accumulative incidence rates | no | 226 | 227 | no | yes | Chinese |
| 2017 | Kuo-Feng Hung[63] | Taiwan | Journal of Ethnopharmacology | Oncology diseases(Gastric Cancer) | Prognosis | Retrospective cohort study | Chinese herbal medicine formulas(unspecified) | Blank control | the risk of mortality and the Kaplan-Meier curve for the survival time | no | 962 | 962 | Catastrophic Illness Patients Database | yes | English |
| 2017 | Fuu-Jen Tsai[64] | Taiwan | Journal of Ethnopharmacology | Nervous system diseases(stroke) | Effect evaluation | Retrospective cohort study | Chinese herbal medicine formulas(unspecified) | Blank control | mortality,overall survival | no | 618 | 618 | National Health Insurance Research Database | no | English |
| 2017 | Kuen-Hau Chen[65] | Taiwan | BMC Complementary and Alternative Medicine | Circulatory system diseases(hypertension) | Prognosis | Retrospective cohort study | Chinese herbal medicine formulas(unspecified) | Blank control | risk of dementia | no | 52365 | 91017 | National Health Insurance Research Database | yes | English |
| 2017 | Shun-Ku Lin[66] | Taiwan | Journal of Ethnopharmacology | Urinary system diseases(Difficult Voiding Symptoms) | Prognosis | Retrospective cohort study | Chinese herbal medicine formulas(unspecified) | Blank control | the rate of patients who received indwelling catheters | no | 2121 | 1861 | National Health Insurance Research Database | no | English |
| 2017 | Mei-Yao Wu[67] | Taiwan | Arthritis Research & Therapy | Immune system diseases(fibromyalgia) | Prognosis | Retrospective cohort study | Acupuncture | Blank control | the risk of coronary heart disease | no | 58899 | 58899 | National Health Insurance Research Database | yes | English |
| 2016 | ZHANG Tong[68] | Mainland China | Chinese Journal of Integrative Medicine | Oncology diseases(Metastatic colorectal cancer) | Effect evaluation | Retrospective cohort study | Chinese herbal medicine formulas(Quxie Capsule)* | Positive control | Overall survival/progression-free survival | no | 110 | 225 | no | yes | English |
| 2016 | Yu-Jun Wang[69] | Taiwan | Evidence-Based Complementary and Alternative Medicine | Oncology diseases(Leukemia) | Prognosis | Retrospective cohort study | Chinese herbal medicine formulas(Unspecified)* | Positive control | Overall survival | no | Pediatric: 292; Adult: 936 | Pediatric: 2063; Adult: 9272 | National Health Insurance Research Database | yes | English |
| 2016 | Yu-Chiang Hung[70] | Taiwan | PLOS ONE | Circulatory system diseases(Atrial Fibrillation) | Prognosis | Prospective cohort study | Chinese herbal medicine formulas(Unspecified)* | Positive control | Occurrence of ischemic stroke | no | 311 | 1715 | National Health Insurance Research Database | yes | English |
| 2016 | Tzung-Yi Tsai[71] | Taiwan | Journal of Ethnopharmacology | Nervous system diseases(vertigo) | Prognosis | Retrospective cohort study | Chinese herbal medicine formulas(Unspecified)* | Positive control | Occurrence of stroke | no | 53203 | 59201 | National Health Insurance Research Database | no | English |
| 2016 | Yuchao L[72] | Mainland China | Academic Journal of Shanghai University of Traditional Chinese Medicine. | Motor system diseases(Sarcopenia) | Effect evaluation | Prospective cohort study | Qigong | Positive control | Life quality:Muscle motion ability | no | 36 | 36 | no | yes | Chinese |
| 2016 | Jun X[73] | Mainland China | China Journal of Traditional Chinese Medicine and Pharmacy. | Motor system diseases(Knee osteoarthritis (swelling phase)) | Effect evaluation | Prospective cohort study | Moxibustion | Other TCM intervention | 1.Life quality:①GPCRND-KOA(Guiding principle of clinical research on new drugs);②WOMAC(The western ontario and mcMaster universities osteoarthritis);2.Data measure:KC(Knee circulation) | no | 30 | 30 | no | yes | Chinese |
| 2016 | San-Yuan Wu[74] | Taiwan | The Journal of Health Care | Urinary system diseases(Urinary stone) | Prognosis | Retrospective cohort study | Chinese herbal medicine formulas（Wu-Ling-San）* | Positive control | The incidence of stone treatment | no | 2382 | 9518 | National Health Insurance Research Database | yes | English |
| 2016 | Min Dai[75] | Mainland China | Evidence-Based Complementary and Alternative Medicine | Oncology diseases(Primary liver cancer) | Prognosis | prospective cohort study | Chinese herbal medicine formulas（Xiao-Chaihu-Decoction）** | Positive control | 1.Life quality:①The evaluation of tumor volume(tumor response);②KPS(Karnofsky performance score);2.Survival related indicators:Survival rate;3.Data measurement:Bodyweight | no | Group1(Xiao-Chaihu):76；Group2(naturopathic treatment):70 | 89 | no | no | English |
| 2016 | Ronald Kiguba[76] | Uganda | BMC Complementary and Alternative Medicine | Others(Not specific) | Adverse events | Prospective cohort study | Single herbs（unspecified） | No control | No access to get data | no | 176 | No access to get data | no | no | English |
| 2016 | HUANG He[77] | Mainland China | CHINA MODERN DOCTOR | Others(perianal abscess) | Effect evaluation | Prospective cohort study | Others(pull-thread and medicated-thread) | Positive control | the cure rate and formation of anal fistula | no | 25 | 25 | no | yes | Chinese |
| 2016 | Liu Zhong-liang[78] | Mainland China | Zhejiang JITCWM | Oncology diseases(advanced non-small cell lung cancer) | Effect evaluation | Retrospective cohort study | Chinese herbal medicine formulas(Yangfei Xiaoji Decotion)* | Positive control | overall survival | no | 51 | 56 | no | yes | Chinese |
| 2016 | ZHANG Ying[79] | Mainland China | JOURNAL OF LIAONING UNIVERSITY OF TCM | Digestive system diseases(liver function) | Effect evaluation | Prospective cohort study | Chinese herbal medicine formulas（Shenfu Injection） | Blank control | ALT、AST | no | 2619 | 11174 | no | yes | Chinese |
| 2016 | Wang Jian-bin[80] | Mainland China | World Science and Technology modernization of Traditional Chinese Medicine and Materia Medica | Oncology diseases(primary liver cancer) | Effect evaluation | Prospective cohort study | Chinese herbal medicine formulas(unspecified) | Blank control | AFP; T lymphocyte subsets;Recurrence and metastasis rate;Overall Survival | distribution of main symptoms of TCM;primary symptom score | 121 | 59 | no | yes | Chinese |
| 2016 | Tzung-Yi Tsai[81] | Taiwan | Journal of Ethnopharmacology | Nervous system diseases(stroke) | Effect evaluation | Prospective cohort study | Chinese herbal medicine formulas(unspecified)(TCM use) | Blank control (non-TCM use) | the risk of stroke incidence | no | 53203 | 59255 | National Health Insurance Research Database | no | English |
| 2016 | Tom Fleischer[82] | Taiwan | Integrative Cancer Therapies | Others(Acute Myeloid Leukemia) | Effect evaluation | Retrospective cohort study | Chinese herbal medicine formulas(unspecified) | Blank control | mortality | no | 249 | 249 | National Health Insurance Research Database | yes | English |
| 2016 | Jui-Ming Liu[83] | Taiwan | Medicine | Oncology diseases(metastatic prostate cancer) | Prognosis | Retrospective cohort study | Chinese herbal medicine formulas(unspecified) | Blank control | mortality,survival rate | no | 730 | 402 | National Health Insurance Research Database | no | English |
| 2016 | Danielle M. Graff[84] | The United States | Pediatric Emergency Care | Nervous system diseases（Pediatric Migraines） | Effect evaluation | Prospective cohort study | Acupuncture | No control | VAS score | no | 19 | 19 | no | no | English |
| 2015 | Yantao Jin[85] | Mainland China | Evidence-Based Complementary and Alternative Medicine | Infectious disease(AIDS) | Prognosis | Retrospective cohort study | Chinese herbal medicine formulas(yi ai kang capsules)* | Positive control | Mortality rate/Cumulative survival | no | 521 | 375 | National TCM HIV Treatment Trial Program | yes | English |
| 2015 | Shanshan Liu[86] | Mainland China | BMC Nephrology | Urinary system diseases(idiopathic membranous nephropathy) | Effect evaluation | Prospective cohort study | Single herbs(Tripterygium wilfordiimultiglycosides)* | Positive control | Remission rate | no | 23 | 30 | no | yes | English |
| 2015 | Der-Shiang Tsai[87] | Taiwan | Journal of Ethnopharmacology | Infectious disease(chronic hepatitis B) | Effect evaluation | Retrospective cohort study | Chinese herbal medicine formulas(Unspecified)* | Positive control | All-cause mortality | no | 506 | 531 | National Health Insurance Research Database | no | English |
| 2015 | Ya Yuwen[88] | Mainland China | Journal of Integrative Medicine | Circulatory system diseases(resistant hypertension) | Effect evaluation | Prospective cohort study | Chinese herbal medicine formulas(11 herbs)* | Positive control | Reductions in blood pressure | TCM symptoms score | 100 | 100 | no | yes | English |
| 2015 | Hsing-Yu Chen[89] | Taiwan | Journal of Ethnopharmacology | Immune system diseases(atopic dermatitis) | Effect evaluation | Retrospective cohort study | Chinese herbal medicine formulas(Unspecified)* | Positive control | Corticosteroid exposure | no | 9012 | 9012 | National Health Insurance Research Database | yes | English |
| 2015 | TANG Zhen-qing[90] | Mainland China | China Journal of Traditional Chinese Medicine and Pharmacy | Nervous system diseases(anxiety symptom of community rehabilitation personnel) | Effect evaluation | Prospective cohort study | Chinese herbal medicine formulas(Jitai Tablets)* | Positive control | Self-Rating Anxiety Scale (SAS) | no | 206 | 180 | no | yes | Chinese |
| 2015 | Dai Guohua[91] | Mainland China | Journal of Traditional Chinese Medicine | Circulatory system diseases(acute myocardial infarction) | Prognosis | Retrospective cohort study | Chinese herbal medicine formulas(Unspecified)* | Positive control | Endpoint events | no | High Exposure Group: 77; Low Exposure Group:58 | 103 | no | yes | Chinese |
| 2015 | DAI Guohua[92] | Mainland China | World Journal of Integrated Traditional and Western Medicine | Circulatory system diseases(Arrhythmia) | Effect evaluation | Retrospective cohort study | Chinese herbal medicine formulas(Formulas for Nourishing Yin Reducing Fire and Calming Down the Mind)* | Positive control | Endpoint events | no | High Exposure Group: 82; Mid Exposure Group: 50; Low Exposure Group:62 | 119 | no | yes | Chinese |
| 2015 | ZHENG Wenguang[93] | Mainland China | Hebei Journal of Traditional Chinese Medicine | Circulatory system diseases(coronary heart disease patients with arrhythmia) | Prognosis | Retrospective cohort study | Chinese herbal medicine formulas(Unspecified)* | Positive control | Endpoint events | no | High Exposure Group: 82; Mid Exposure Group: 50; Low Exposure Group:62 | 119 | no | yes | Chinese |
| 2015 | WU Maolin[94] | Mainland China | Western Journal of Traditional Chinese Medicine | Oncology diseases(Advanced Esophageal Cancer) | Effect evaluation | Prospective-Retrospective cohort study | Chinese herbal medicine formulas(Modified BanXia XieXin Tang)* | Positive control | Improvement ofeating choking sense/Karnofsky Performance Status/Quality of life/Overall survival | no | 35 | 35 | no | no | Chinese |
| 2015 | Meili Y[95] | Mainland China | Chinese Journal of Integrative Medicine on Cardio-/Cerebrovascular Disease. | Circulatory system diseases(Angina pectoris) | Prognosis | Prospective cohort study | Others (Tianjiu) | Blank control | 1.Efficacy；2.Life quality:Likert scale | no | 110 | 110 | no | yes | Chinese |
| 2015 | Jun X[96] | Mainland China | Journal of Traditional Chinese Medicine. | Motor system diseases(Lumbar disc herniation) | Effect evaluation | Prospective cohort study | Moxibustion | Other TCM intervention | Life quality:①M-JOA(Modified Japanese orthopaedic association low back pain score scale);②VAS(Visual analogue score) | no | 30 | 30 | no | yes | Chinese |
| 2015 | Jingru L[97] | Mainland China | World Science and Technology/Modernization of Traditional Chinese Medicine and Materia Medica. | Immune system diseases(AIDS diarrhea) | Effect evaluation | Prospective cohort study | Moxibustion | Blank control | Life quality:Time of symptom improvement | TCM syndrome score | 14 | 22 | no | yes | Chinese |
| 2015 | Zhenping L[98] | Mainland China | Journal of Shanxi College of Traditional Chinese Medicine. | Gynecology diseases(Hyperplasia of mammary glands) | Effect evaluation | Prospective cohort study | Acupuncture | Other TCM intervention | Efficacy | no | 415 | 106 | no | no | Chinese |
| 2015 | Xiong Jun[99] | Mainland China | Acupuncture Research. | Gynecology diseases(Primary dysmenorrhea) | Effect evaluation | Prospective cohort study | Moxibustion | Other TCM intervention | Life quality:①MPQ(McGill pain questionaire,McGill);②CMSS(Cox menstrual symptom scale,Cox) | no | 35 | 35 | no | yes | Chinese |
| 2015 | FAN Junming[100] | Mainland China | Chinese Acupuncture & Moxibustion. | Nervous system diseases(Cerebral infarction) | Effect evaluation | Retrospective cohort study | Acupuncture* | Positive control | Life quality:Lovertt | no | 110 | 110 | no | yes | Chinese |
| 2015 | Rui Liu[101] | Mainland China | Evidence-Based Complementary and Alternative Medicine | Oncology disease(Small cell lung cancer) | Effect evaluation | Prospective cohort study | Chinese herbal medicine formulas（unspecified） | No control | 1.Life quality:Function Assessment;2.Survival related indicators:Survival time. | TCM syndromes score | 28 | no | no | yes | English |
| 2015 | Chih-Wen Chiu[102] | Taiwan | BMC Complementary and Alternative Medicine | Nervous system diseases(Dizziness and vertigo) | Effect evaluation | Prospective cohort study | Acupuncture | Positive control | 1.Bioindicator:HRV(Heart rate variability);2.Life quality:①DHI(Dizziness Handicap Inventory);②VAS(Visual Analog Scale);③Safety and length of stay. | no | 37 | 23 | no | yes | English |
| 2015 | MAO Jun[103] | Mainland China | Chin J of Clinical Rational Drug Use | Nervous system (cerebral infarction) | Prognosis | Prospective cohort study | Single herbs(Tea)(occasionally drink tea and often drink tea) | Blank control(never drink tea) | FPG、TG、TC、Scr | no | 31381 | 65223 | no | no | Chinese |
| 2015 | SHI Lin[104] | Mainland China | Chinese Clinical Oncology | Oncology diseases(breast cancer) | Effect evaluation | Prospective cohort study | Chinese herbal medicine formulas(unspecified) ** | Two and above controls(Control group1：Positive control；Control group2：Blank control) | accumulative disease-free survival (DFS) rates，accumulative overall survival (OS) rates，median DFS and OS；Safety indicator | no | 150 | 204 | no | yes | Chinese |
| 2015 | Fabian aus dem siepen[105] | Germany | Drug Design, Development and Therapy | Circulatory system diseases(wild-type transthyretin amyloidosis) | Effect evaluation | Prospective cohort study | Single herbs(Green tea extract)(GTE capsules) | No control | cMRI;Blood;Echocardiography | no | 25 | 25 | no | yes | English |
| 2014 | Hsienhsueh Elley Chiu1[106] | Taiwan | PLOS ONE | Nervous system diseases(stroke) | Prognosis | Retrospective cohort study | Acupuncture(Unspecified)* | Positive control | Average re-admission duration | no | 238 | 238 | National Health Insurance Research Database | no | English |
| 2014 | Yantao Jin[107] | Mainland China | The American Journal of Chinese Medicine | Infectious disease(AIDS) | Prognosis | Retrospective cohort study | Chinese herbal medicine formulas(Unspecified)* | Positive control | Mortality rate/Cumulative survival | no | 1442 | 1787 | NTCMTP database/Epidemiological database/Treatment database of the Chinese CDC | yes | English |
| 2014 | Wei Zhang[108] | Mainland China | The American Journal of Chinese Medicine | Oncology diseases(hepatoma) | Effect evaluation | Retrospective cohort study | Single herbs(cantharidins)* | Positive control | Criteria for the codification of tumor response evaluation by World Health Organization | no | 96 | 95 | no | no | English |
| 2014 | Hai-Lu Zhao[109] | Mainland China | The American Journal of Chinese Medicine | Infectious disease(AIDS) | Prognosis | Prospective cohort study | Chinese herbal medicine formulas(16-herb formulas)* | Positive control | Mortality rate/Cumulative survival | no | 165 | 220 | no | yes | English |
| 2014 | Yuan-Wen Lee[110] | Taiwan | Cancer | Oncology diseases(advanced breast cancer) | Prognosis | Retrospective cohort study | Chinese herbal medicine formulas(Unspecified)* | Positive control | All-cause mortality | no | 115 | 614 | National Health Insurance Research Database | yes | English |
| 2014 | Zhu-Qing Ji[111] | Mainland China | Asian Pacific Journal of Cancer Prevention, | Oncology diseases(Non Small-Cell Lung Cancer) | Adverse event | Prospective cohort study | Chinese herbal medicine formulas(Brucea javanica and Cantharidin)* | Positive control | Adverse event | no | Brucea javanica group: 18; Cantharidin group: 18; Brucea javanica and Cantharidin group: 16 | 18 | no | yes | English |
| 2014 | Yu-Chiang Hung[112] | Taiwan | Complementary and Alternative Medicine | Respiratory system diseases(asthma) | Effect evaluation | Retrospective cohort study | Chinese herbal medicine formulas(Unspecified)** | Positive control | Medical visit rate/Visit frequency/Cost paid | no | Single TCM group: 406; Integrative OHQI TCM group: 9 | 12435 | Longitudinal Health Insurance Database 2000 | no | English |
| 2014 | ZHU Huayu[113] | Mainland China | LISHIZHEN MEDICINE AND MATERIA MEDICA RESEARCH | Gynecological diseases(Granulomatous mastitis) | Effect evaluation | Retrospective cohort study | Acupuncture(Rad-hot needle therapy)* | Positive control | Harris evaluation criteria | Clinical Criteria for Diagnosis of Surgical Syndrome in TCM | 40 | 54 | no | yes | Chinese |
| 2014 | WANG Zhiying[114] | Mainland China | Journal of Nanjin University of TCM | Respiratory system diseases(Chronic Persistent Asthma) | Effect evaluation | Prospective cohort study | Chinese herbal medicine formulas(Wenyang Huatan Fang/Qingyang Huatan Fang) | Other Traditional Chinese Medicine intervention(Guben Kechuan tang/Maiwei Dihuang tang) | Efficiency rate/IgE/ECP/IL-4/IFN-yin | no | 121 | 81 | no | yes | Chinese |
| 2014 | XU Danping[115] | Mainland China | Chinese Journal of Gerontology | Circulatory system diseases(coronary heart disease) | Effect evaluation | Prospective cohort study | Chinese herbal medicine formulas(Deng Tietao's formulas for coronary heart disease)* | Positive control | Angina pectoris score/Survival treatment angina pectoris pain scale score/Mortality rate/Endpoint events | no | 333 | 204 | no | yes | Chinese |
| 2014 | QIU Yiwen[116] | Mainland China | Journal of Guangzhou University of Traditional Chinese Medicine | Oncology diseases(hepatocellular carcinoma) | Prognosis | Retrospective cohort study | Chinese herbal medicine formulas(Formula of Diagnosis and treatment of primary liver cancer)** | Positive control | Median survival time/Survival rate | no | TCM group: 241; TCM with WM group: 195 | 53 | no | yes | Chinese |
| 2014 | SUN Zhen[117] | Mainland China | JOURNAL OF LIAONING UNIVERSITY OF TCM | Oncology diseases(hepatocellular carcinoma) | Prognosis | Retrospective cohort study | Chinese herbal medicine formulas(Ruanjian Hugan tablets & Cinobufotalin injection)** | Positive control | Disease free survival/Overall survival | no | TCM group: 53; TCM with IT group: 20 | TACE group: 80; SO group: 8 | no | yes | Chinese |
| 2014 | Chun-Chuan Shih[118] | Taiwan | plosone | Nervous system diseases(Stroke) | Prognosis | Retrospective cohort study | Acupuncture* | Positive control | Proportion of new-onset stroke events | no | 7409 | 29636 | National Health Insurance Research Database | no | English |
| 2014 | [119] | Mainland China | Jiangsu Journal of Traditional Chinese Medicine | Oncology diseases(Gastric Cancer) | Effect evaluation | Prospective cohort study | Chinese herbal medicine formulas(Yiqi Huayu Jiedu Decoction)* | Positive control | recurrence and metastasis rate；Disease-free survival probability | no | 201 | 196 | no | yes | Chinese |
| 2014 | Zhou Dai-han[120] | Mainland China | WORLD CHINESE MEDICINE | Oncology diseases(non-small cell lung cancer) | Effect evaluation | Prospective cohort study | Two and above TCM interventions:Qingjin Desheng tablets,ginseng capsule、Chinese herbal decoction based on Yiqi Chutan method and Acupuncture | Positive control | median survival time;time to progress | no | 167 | 148 | no | yes | Chinese |
| 2014 | Shuo-Meng Wang[121] | Taiwan,China | PLOS ONE | Oncology diseases（ Urinary Tract Cancer） | Adverse reaction | Retrospective cohort study | Single herbs(Containing Aristolochic Acid) | blank conttrol | the occurrence rate of urinary tract cancers | no | 320 | 38675 | National Health Insurance Research Database | yes | English |
| 2013 | Youfu Ke[122] | Hong Kong | Complementary Therapies in Medicine | Circulatory system diseases(Essential hypertension) | Effect evaluation | Prospective cohort study | Chinese herbal medicine formulas(Wuling powder and modified tianma gouteng decoction) | Self-control | New York Heart Association heart function classification/heart rate/blood pressure/6-min walking test | no | 72 | 72 | no | no | English |
| 2013 | Yueh-Hsiang Huang[123] | Taiwan | Integrative Cancer Therapies | Oncology diseases(head and neck cancer) | Effect evaluation | Retrospective cohort study | Chinese herbal medicine formulas(Unspecified)* | Positive control | Body weight/ M.D. Anderson Symptom Inventory | no | 69 | 74 | no | yes | English |
| 2013 | WANG Jing[124] | Mainland China | Guiding Journal of Traditional Chinese Medicine and Pharmacy | Others（Sepsis） | Effect evaluation | Prospective cohort study | Chinese herbal medicine formulas(Qi-Clearing Construction-Cooling Decoction)* | Positive control | 28day mortality rate/APACHE score/serum HMGB1 concentration | no | 30 | 30 | no | yes | Chinese |
| 2013 | LIU Guoqing[125] | Mainland China | Chinese Journal of Health Care Nutrition | Others（Tibial fracture） | Effect evaluation | Retrospective cohort study | Chinese herbal medicine formulas(Taohong Siwu Jiegu formulas)* | Positive control | Fracture healing rate/Fracture healing time/Complications | no | 39 | 50 | no | yes | Chinese |
| 2013 | ZHU Weirong[126] | Mainland China | Chin J Integr Trad West Med Dig | Oncology diseases(pancreatic cancer) | Prognosis | Retrospective cohort study | Chinese herbal medicine formulas(Fuzheng Quxie Regimen)* | Positive control | Overall survival/Median survival time | no | 20 | 26 | no | no | Chinese |
| 2013 | QIU Ruijin[127] | Mainland China | Journal of Changchun University of Traditional Chinese Medicine | Oncology diseases(mammary cancer) | Effect evaluation | Prospective cohort study | Chinese herbal medicine formulas(Shuganyishen formulas)* | Positive control | EORTC quality of life measurement scale QLQ-C30 (V3.0) chinese version | no | 67 | 59 | no | yes | Chinese |
| 2013 | Cong Z[128] | Mainland China | Journal of Beijing University of Traditional Chinese Medicine · Clinical Medicine. | Others(Fatigue-predominant sub-health) | Effect evaluation | Prospective cohort study | Qigong | Blank control | No | TCM symptoms questionnaires score | 62 | 67 | no | yes | Chinese |
| 2013 | Yin Dongfeng[129] | Mainland China | Modern Oncology. | Oncology disease(Breast cancer) | Prognosis | Prospective cohort study | Chinese herbal medicine formulas(Ruyanning) | Positive control | Survival related indicators:①Time of endocrine therapy;②Overall survival;③Survival time after recurrence | no | 33 | 17 | no | yes | Chinese |
| 2013 | James W. Ochi[130] | The United States | Pediatric Otorhinolaryngology | Pediatric disease(Tonsillectomy pain) | Effect evaluation | Retrospective cohort study | Acupuncture | No control | Life quality:Pain level | no | 31 | no | no | yes | English |
| 2013 | June-Seek Choi[131] | Korea | Planta Med | Reproductive system diseases(Fetal abnormalities or stillbirths) | Adverse events | Prospective cohort study | Single herbs( Licorice (Glycyrrhiza uralensis)) | Blank control | 1.Data measurement:①Birth weight (grams);②Birth length (cm);③Head circumfer- ence (cm);④1-min and 5-min postnatal Apgar scores;2.Fetal and Neonatal Outcomes( incidence of stillbirths, gestational age at birth (weeks)and incidence of meconium staining, gross malformations, and neonatal jaundice) | no | 185 | 370 | no | yes | English |
| 2013 | ZHOU Yu-qian[132] | Mainland China | Chinese Journal of Integrated Traditional and Western Medicine | Nervous system diseases(multiple sclerosis) | Effect evaluation | Prospective cohort study | Chinese herbal medicine formulas(unspecified)* | Positive control | the recurrence intervals and the yealy average recurrence times | no | 14 | 21 | no | no | Chinese |
| 2013 | HUANG Qi[133] | Mainland China | Journal of Emergency in Traditional Chinese Medicine | Circulatory system diseases(coronary heart disease ventricular premature beat) | Effect evaluation | Retrospective cohort study | Chinese herbal medicine formulas(Maianning mixture)* | Positive control | effect of dynamic electrocardiogram;rehospitalization rates | the TCM syndrome score | 51 | 48 | no | no | Chinese |
| 2013 | Zhu Dong[134] | Mainland China | World Science and Technology modernization of Traditional Chinese Medicine and Materia Medica | Motor system diseases(cervical spondylotic myelopathy) | Effect evaluation | Ambispective Cohort Study | Chinese herbal medicine formulas( based on Shengyu Decoction) | TCM intervention (dose control) | the 40-score and JOA total score | no | 44 | 36 | no | yes | Chinese |
| 2013 | ZHAO Yuan[135] | Mainland China | Practical Journal of Clinical Medicine | Motor system diseases(knee degenerative osteoarthritis) | Effect evaluation | Prospective cohort study | Two and above TCM interventions（acupuncture、massage、CHMF） | No control | knee osteoarthritis score | no | 45 | 45 | no | no | Chinese |
| 2012 | ZHAO Xiao-feng[136] | Mainland China | Journal of Traditional Chinese Medicine | Nervous system diseases(stroke) | Prognosis | Retrospective cohort study | Acupuncture("Xing Nao Kai Qiao" acupuncture use≥24 times) | Traditional Chinese Medicine intervention (dose control)：Acupuncture("Xing Nao Kai Qiao" acupuncture use＜24 times) | Mortality rate/vascular event | no | 239 | 166 | no | yes | English |
| 2012 | DUAN Wen-hui[137] | Mainland China | Chin J Integr Med | Circulatory system diseases(acute myocardial infarction) | Effect evaluation | Prospective cohort study | Chinese herbal medicine formulas(Unspecified)* | Positive control | Death/myocardial infarction (MI)/revascularization/stroke/rehospitalization/heart dysfunction | TCM symptoms score | 169 | 165 | no | yes | English |
| 2012 | Hu XY[138] | Mainland China | Journal of Chinese Integrative Medicine | Infectious disease(chronic hepatitis B) | Effect evaluation | Prospective cohort study | Chinese herbal medicine formulas(Qinggan formulas)* | Positive control | Cumulative survival/Biochemical and coagulation indicators | Classification of common symptoms of viral hepatitis | 68 | 37 | no | yes | Chinese |
| 2012 | ZHANG Tao[139] | Mainland China | Chinese Journal of Integrated Traditional and Western Medicine | Infectious disease(chronic hepatitis B) | Effect evaluation | Prospective cohort study | Chinese herbal medicine formulas（Shenxian Yiganling）* | Positive control | ALT levels/HBV DNA levels | no | 71 | 80 | no | no | Chinese |
| 2012 | ZHANG Liling[140] | Mainland China | Nei Mongol Journal of Traditional Chinese Medicine | Pediatric disease(hand-foot-and-mouth disease) | Effect evaluation | Retrospective cohort study | Chinese herbal medicine formulas(Unspecified)** | Positive control | Time of symptom disappear | no | TCM group: 31; TCM with WM group: 52 | 34 | no | no | Chinese |
| 2012 | ZHANG Guoliang[141] | Mainland China | Journal of Traditional Chinese Medicine | Pediatric disease(hand-foot-and-mouth disease) | Effect evaluation | Prospective cohort study | Chinese herbal medicine formulas(Unspecified)** | Positive control | Time of symptom disappear | no | TCM group: 409; TCM with WM group: 304 | 124 | no | yes | Chinese |
| 2012 | WANG Zhiying[142] | Mainland China | Journal of Nanjin University of TCM | Respiratory system diseases(Asthma) | Effect evaluation | Prospective cohort study | Chinese herbal medicine formulas(Wenyang Huatan Fang/Qingyang Huatan Fang) | Other Traditional Chinese Medicine intervention(Guben Kechuan tang/Maiwei Dihuang tang) | Asthma control test score/Efficiency rate/Pulmonary function | TCM symptoms score | 121 | 81 | no | yes | Chinese |
| 2012 | WAN Chanjun[143] | Mainland China | Beiiing Journal of Traditional Chinese Medicine | Circulatory system diseases(cardiac syndrome X) | Effect evaluation | Retrospective cohort study | Chinese herbal medicine formulas(Liqi Kuanxiong Huoxue formulas)* | Positive control | Changes in angina pectoris/Endpoint events | TCM symptoms score | 19 | 16 | no | yes | Chinese |
| 2012 | Adrian White[144] | The United Kingdom | Acupunct Med | Motor system diseases(Knee pain) | Prognosis | Retrospective cohort study | Acupuncture | No control | Life quality:MYMOP(Measure Yourself Medical Outcome Profile) | no | 90 | no | no | yes | English |
| 2012 | GAO Hui[145] | Mainland China | Chinese Journal of Integrated Traditional and Western Medicine | Urinary system diseases(progress of chronic renal failure) | Effect evaluation | Prospective cohort study | Chinese herbal medicine formulas(unspecified)* | Positive control | Biological indicator:HB，ALB，SCr，eGFR，reciprocal slope of serum creatinine and endpoint events | no | 59 | 51 | no | yes | Chinese |
| 2012 | CHEN Xiao-zhong[146] | Mainland China | HUNAN JOURNAL OF TRADITIONAL CHINESE MEDICINE | Digestive system diseases（Chronic hepatitis B） | Effect evaluation | Prospective cohort study | Chinese herbal medicine formulas(unspecified)* | Positive control | Biological indicator:1.the normalization rates of ALT 2.the patients proportion of HBV-DNA below the detection value 3.the HBeAg negative conversion rates 4.the HBeAg seroconversion rates;The incidence of adverse events | no | 68 | 69 | no | yes | Chinese |
| 2012 | LI Qing[147] | Mainland China | Chinese Journal of Integrated Traditional and Western Medicine | Endocrine system diseases(diabetic nephropathy) | Effect evaluation | Prospective cohort study | Chinese herbal medicine formulas(unspecified)* | Positive control | the incidence of endpoint events and secondary outcome measures;the liver function，blood routines and ECG. | no | 116 | 54 | no | yes | Chinese |
| 2012 | Julia A. Montague[148] | Singapore | Cancer Causes Control | Oncology diseases(prostate cancer) | Prognosis | Prospective cohort study | Single herbs( green tea-only,black tea-only rinkers,both tea types) | blank control(non-tea) | prostate cancer risk | no | 17988 | 8859 | Singapore Cancer Registry database | no | English |
| 2011 | LIU Qingchi[149] | Mainland China | Chinese Journal of Experimental Traditional Medical Formulae | Others(Chronic Aplastic Anemia) | Effect evaluation | Prospective cohort study | Chinese herbal medicine formulas(Longteng Shengxue Pill)* | Positive control | Morphological changes of bone marrow cells | no | 60 | 60 | no | no | Chinese |
| 2011 | GUAN Ruodan[150] | Mainland China | Guangdong Medical Journal | Oncology diseases(mammary cancer) | Effect evaluation | Retrospective cohort study | Single herbs(Huaier Granule)* | Positive control | Recurrence and metastasis o f HCC/Disease free survival rate/Survival rate | no | 77 | 88 | no | yes | Chinese |
| 2011 | LIU Jing[151] | Mainland China | Journal of Clinical Medicine in Practice | Oncology diseases(Colorectal cancer) | Effect evaluation | Prospective cohort study | Chinese herbal medicine formulas(Jianpi Compoud Recipe)* | Positive control | Overall survival/Quality of Life Score | no | 37 | 29 | no | yes | Chinese |
| 2011 | LU Wenping[152] | Mainland China | International Journal of Traditional Chinese Medicine | Oncology diseases(mammary cancer) | Effect evaluation | Prospective cohort study | Chinese herbal medicine formulas(Shuganyishen formulas)* | Positive control | Time to progress/Overall survival/Quality of life | no | 126 | 100 | no | no | Chinese |
| 2011 | Yan L[153] | Mainland China | Journal of Beijing University of Traditional Chinese Medicine. | Others(Fatigue-predominant sub-health) | Effect evaluation | Prospective cohort study | Qigong | Blank control | Life quality:SF-36(The Medical Outcomes Study Short Form) | no | 62 | 67 | no | yes | Chinese |
| 2011 | Jin gang D[154] | Mainland China | Chinese Journal of Basic Medicine in Traditional Chinese Medicine. | Others(Fatigue-predominant sub-health) | Effect evaluation | Prospective cohort study | Qigong | Blank control | Life quality:Basic physical ability | no | 62 | 67 | no | yes | Chinese |
| 2011 | Jianling M[155] | Mainland China | Modern Journal of Integrated Traditional Chinese and Western Medicine. | Circulatory system diseases(Hypertention) | Effect evaluation | Prospective cohort study | Acupuncture* | Positive control | Bioindicator:Blood pressure(①SBP；②DBP；③MAP | no | 37 | 105 | no | no | Chinese |
| 2011 | ZHONG Run-fen[156] | Mainland China | Chinese Acupuncture & Moxibustion. | Nervous system diseases(Spontaneous facial paralysis) | Effect evaluation | Prospective cohort study | Two and above TCM interventions | Other TCM intervention | Life quality:①HB(House-Brackmann)；②FDI(Facial disable index) | no | 59 | 21 | no | no | Chinese |
| 2011 | Michael McCulloch[157] | The United States | Integrative Cancer Therapies | Oncology disease(Colon cancer) | Prognosis | Retrospective cohort study | Chinese herbal medicine formulas（ Pan-Asian medicine + vitamins (PAM+V)）* | Positive control | Survival related indicators:Survival rates | no | 193 | 11,678 | California Cancer Registry (CCR; www.ccrcal.org) and Kaiser Permanente Northern California (KPNC) Division of Research Cancer Registry (www.dor.kaiser.org) | yes | English |
| 2011 | YANG Chang-kun[158] | Mainland China | Journal of Changchun University of Traditional Chinese Medicine | Digestive system diseases（Chronic hepatitis B） | Effect evaluation | Retrospective cohort study | Chinese herbal medicine formulas(unspecified) | Blank control | Incidence of cirrhosis | no | 65 | 155 | no | no | Chinese |
| 2010 | Pei-Jen Chang[159] | Taiwan | International Journal of Nursing Studies | Gynecological diseases（postpartum women） | Effect evaluation | Prospective cohort study | Chinese herbal medicine formulas（Sheng-Hua-Tang） | Traditional Chinese Medicine intervention (dose control) | 36-item Short-Form | no | Within 1 month only: 15009; Within 1 month and later:351 | No use:3173 | Taiwan national birth register | yes | English |
| 2010 | ZHANG Guoliang[160] | Mainland China | Anhui Medical Journal | Pediatric disease(hand-foot-and-mouth disease) | Effect evaluation | Prospective cohort study | Chinese herbal medicine formulas(Unspecified)** | Positive control | Time of symptom disappear | no | TCM group: 85; TCM with WM group: 220 | 42 | no | yes | Chinese |
| 2010 | LIU Zhaoyang[161] | Mainland China | Chinese J T rad Med T raum & Orthop | Nervous system diseases(Women with Convalescent Cerebral Concussion) | Effect evaluation | Prospective cohort study | Chinese herbal medicine formulas(Modified Xiaoyaosan) | Other Traditional Chinese Medicine intervention(Bugan tang/Kebaolisu tang) | Simplified SF-36 scale | Chinese criteria of diagnosis and the rapeutic effect | 23 | 45 | no | no | Chinese |
| 2010 | HUANG Wei[162] | Mainland China | Chin J Bases Clin General Surg | Oncology diseases(hepatocellular carcinoma) | Effect evaluation | Retrospective cohort study | Single herbs(Huaier Granule)* | Positive control | Immune rejection/Recurrence and metastasis o f HCC/Disease free survival rate/Survival rate | no | 28 | 56 | no | yes | Chinese |
| 2010 | Xiaoping Z[163] | mainland China | Li Shi Zhen Medicine and Materia Medica Research. | Respiratory system diseases(Cold headache) | Effect evaluation | Prospective cohort study | Two and above TCM interventions | Other TCM intervention | Life quality：VAS(Visual analogue score) | no | Group1(Scrapping):45;Group2(Moving cupping):48 | 34 | no | no | Chinese |
| 2010 | Fan Yuling[164] | mainland China | Medical Information. | Respiratory system diseases(Post-infectious cough) | Effect evaluation | Prospective cohort study | Acupuncture | Other TCM intervention | Recovery time | no | 35 | 54 | no | no | Chinese |
| 2010 | WU Shi-yan[165] | Mainland China | Journal of Traditional Chinese Medical Literature | Oncology diseases(liver metastasis from colorectal cancer) | Effect evaluation | Prospective cohort study | Chinese herbal medicine formulas(Ling Yaoxing's experience in treating cancer) | Positive control | survival rates | no | 53 | 56 | no | yes | Chinese |
| 2010 | ZHU Hui[166] | Mainland China | Chinese Journal of Integrated Traditional and Western Medicine | Circulatory system diseases（coronary heart disease） | Effect evaluation | Prospective cohort study | Chinese herbal medicine formulas(Shexiang Baoxin Pill)* | Positive control | Survivor-related indicator:1.Clinical events2.Deaths | no | 93 | 90 | no | yes | Chinese |
| 2010 | GUAN Jia-hui[167] | Mainland China | ONCOLOGY PROGRESS | Oncology diseases(After radical resection of colorectal cancer) | Effect evaluation | Prospective cohort study | Two and above TCM interventions(Chinese herbal medicine formulas(unspecified)+any of Sinopus tablet, quxie capsule, pingxiao capsule)* | Positive control | Recurrence and metastasis rate | no | 97 | 98 | no | yes | Chinese |
| 2009 | Sang-Wook Yi[168] | Korea | THE JOURNAL OF ALTERNATIVE AND COMPLEMENTARY MEDICINE | Others(≥55 years old healthy volunteers) | Prognosis | Prospective cohort study | Single herbs(Ginseng) | Traditional Chinese Medicine intervention (dose control) | Mortality rate/Cancer-specific mortality | no | infrequent user: 2840; frequent user: 840 | nonuser: 2602 | no | no | English |
| 2009 | CHEN Jian[169] | Mainland China | China Journal of Traditional Chinese Medicine and Pharmacy | Pediatric disease(hand-foot-and-mouth disease) | Effect evaluation | Retrospective cohort study | Chinese herbal medicine formulas(Yinqiao san and Ganluxiaodu dan/Qingwenbaidu yin)* | Positive control | Time of symptom disappear | no | 91 | 89 | no | no | Chinese |
| 2009 | Richard Simcock[170] | The United Kingdom | Acupunct Med | Others(Radiation induced xerostomia) | Effect evaluation | Prospective cohort study | Acupuncture | No control | 1.Life quality:①EORTC QLQ-C30 (version 3.0);②EORTC QLQ H&N 35;2.Data measurement:Mouth salivation measure | no | 12 | no | no | yes | English |
| 2009 | Hsiao-Yu Yang[171] | Taiwan | J Epidemiol | Oncology disease(Cancers of the kidney and other urinary organs) | Adverse events | Retrospective cohort study | Single herbs（unspecified） | Blank control | Survival related indicators:SMR(Standardized mortality ratio) | no | 6548 | 88,289 | National Mortality Registry Database;database of the Bureau of Labor Insurance | no | English |
| 2009 | WU Huan-lin[172] | Mainland China | Jilin Journal of Traditional Chinese Medicine | Circulatory system diseases(coronary artery bypass graft) | Effect evaluation | Retrospective cohort study | Two and above TCM interventions(Denglao Guanxin Recipe;Denglao Guanxin Capsule)* | Positive control | Survivor-related indicator:1.The incidence of recurrent chest pain 2.Rehospitalization rates 3.The incidence of epigastric discomfort | no | 28 | 21 | no | yes | Chinese |
| 2009 | WU Huan-lin[173] | Mainland China | China Journal of Modern Medicine | Circulatory system diseases（PCI treatment of coronary heart disease） | Effect evaluation | Prospective cohort study | Chinese herbal medicine formulas(Denglao Guanxin Recipe)* | Positive control | Survivor-related indicator:1.Recurrence of cardiovascular events 2.Gastrointestinal complications | no | 48 | 248 | no | yes | Chinese |
| 2009 | GE Jian-zhong[174] | Mainland China | Chinese Journal of Gerontology | Oncology diseases(after radical resection of elderly colorectal cancer) | Effect evaluation | Prospective cohort study | Chinese herbal medicine formulas(unspecified)* | Positive control | Survivor-related indicator:1.Recurrence and metastasis rate 2.Recurrence and metastasis time | no | 98 | 104 | no | no | Chinese |
| 2009 | Ikue Watanabe[175] | Japan | Am J Clin Nutr | Respiratory system diseases(pneumonia) | Effect evaluation | Prospective cohort study | Single herbs(Green tea)(1-2cups/d,3-4cups/d,and>5cups/d) | TCM intervention (dose control)(Green tea)(<1cups/d) | death from pneumonia | no | 29920 | 10652 | the Ohsaki National Health Insurance (NHI) beneficiaries | no | English |
| 2008 | J. A. Olalde[176] | Venezuela | PHYTOTHERAPY RESEARCH | Endocrine system diseases(diabetic foot) | Effect evaluation | Retrospective cohort study | Chinese herbal medicine formulas(Circulat)^*^ | Positive control | University of Texas Diabetic Wound Classification Grade | no | 174 | No access to get data | no | no | English |
| 2008 | Mohammad Heidary[177] | Iran | Urology Journal | Urinary system diseases(infertility) | Effect evaluation | Prospective cohort study | Single herbs(saffron) | Self-control | Semen analysis | no | 52 | 52 | no | no | English |
| 2008 | HUANG Xuerong[178] | Mainland China | Herald of Medicine | Nervous system diseases(Fever) | Adverse event | Retrospective cohort study | Single herbs(Puerarin Injection) | Other Traditional Chinese Medicine intervention(Puerarin Injection of other brand) | Incidence of fever | no | 650 | 762 | no | no | Chinese |
| 2008 | LIANG Bi-yan[179] | Mainland China | Chinese Journal of Information on TCM. | Oncology disease(Colorectal cancer) | Prognosis | Prospective cohort study | Chinese herbal medicine formulas（unspecified）* | Positive control | Survival related indicators:①Relapse rates;②Disease-free survival rates | no | 84 | 78 | no | yes | Chinese |
| 2008 | YANG Yu-fei[180] | Mainland China | Chinese Journal of Integrative Medicine | Oncology disease(Colorectal cancer after radical operation) | Prognosis | Prospective cohort study | Chinese herbal medicine formulas（Sijunzi Decoction, Chaihu Shugan Powder or Bazhen Decoction etc.and a traditional patented drug, such as Huachansu Tablet, Quxie Capsule or Pingxiao Capsule.)* | Positive control | Survival related indicators:①Relapse rates;②Metastasis rates;③Time of relapse;④Time of metastasis. | no | 98 | 104 | no | yes | English |
| 2008 | S Jena[181] | Germany | Cephalalgia | Nervous system diseases(Headache) | Effect evaluation | Prospective cohort study | Acupuncture | Positive control | Life quality：①Number of days with headache per month;②SF-36(The Medical Outcomes Study Short Form);③The use of cointerventions | no | 11874 | 1693 | no | no | English |
| 2007 | Jean-Francois Dartigues[182] | France | JAGS | Nervous system diseases(dementia) | Prognosis | Prospective cohort study | Single herbs(Ginkgo biloba)* | Positive control | Dementia/Mortality | no | 225 | 888 | no | yes | English |
| 2007 | Sun Chang[183] | Mainland China | Journal of New Chinese Medicine. | Motor system diseases(Periarthritis humeroscapularis) | Effect evaluation | Prospective cohort study | Acupuncture | Other TCM intervention | 1.Efficacy ；2.Life quality:PRI(Pain rating index) | no | 79 | 69 | no | no | Chinese |
| 2007 | Kar Seng Lim[184] | Singapore | Annals Academy of Medicine | Others(Chronic venous leg ulcer) | Adverse events | Prospective cohort study | Chinese herbal medicine formulas（unspecified)* | Positive control | Bioindicator:Positive PT reading | no | 17 | 24 | no | no | English |
| 2007 | Can-Lan Sun[185] | Singapore | Carcinogenesis | Oncology diseases(colorectal cancer) | Prognosis | Prospective cohort study | Single herbs(Green tea and black tea consumption)(Monthly/Weekly/Daily) | Blank control(non-tea) | incident cancer cases and deaths | no | 47217 | 25292 | Singapore Chinese Health Study | yes | English |
| 2007 | Farin Kamangar[186] | MailandChina | Cancer Epidemiol Biomarkers Prev | Oncology diseases(Gastric Cancer) | Prognosis | Prospective cohort study | Single herbs(Ginseng) | Blank control(no ginseng use) | gastric cancer risk | no | 21318 | 52134 | no | yes | English |
| 2006 | Li Guangxi[187] | Mainland China | China's Naturopathy. | Respiratory system diseases(Asthma) | Effect evaluation | Prospective cohort study | Acupoint application | No control | Efficacy | no | 893 | no | no | no | Chinese |
| 2006 | SHARON K. HULL[188] | The United States | THE JOURNAL OF ALTERNATIVE AND COMPLEMENTARY MEDICINE | Others(Not specific) | Prognosis | Prospective cohort study | Acupuncture | No control | Life quality:①SF-36(The Medical Outcomes Study Short Form);②MYMOP questionnaire;③Global clinical change,④Patient satisfaction | no | 110 | no | no | yes | English |
| 2006 | LUO Lin [189] | Mainland China | Chinese Journal of Integrated Traditional and Western Medicine | Oncology diseases(Postoperative recurrence and metastasis of colorectal cancer) | Effect evaluation | Prospective cohort study | Chinese herbal medicine formulas(Fuzheng Capsule and Quxie Capsule) | Blank control | Survivor-related indicator:1.Recurrence and metastasis rate 2.Recurrence and metastasis time 3.Incidence of adverse events | no | 53 | 48 | no | no | Chinese |
| 2005 | JOSEPH T.F. LAU[190] | HongKong | THE JOURNAL OF ALTERNATIVE AND COMPLEMENTARY MEDICINE | Infectious disease(SARS) | Effect evaluation | Prospective cohort study | Chinese herbal medicine formulas（ Sang Ju Yin and Yu Ping Feng San ） | Positive control | 1.Efficacy of SARS prevention;2.Life quality:VAS(Visual analogue scale). | Chinese version of the Short Form-36 (SF-36) score | 1063 | 36,111 | Hospital Authority’s SARS registry database | yes | English |
| 2005 | Motohiro Inoue[191] | Japan | ACUPUNCTURE IN MEDICINE | Nervous system diseases(Radicular sciatica) | Effect evaluation | Prospective cohort study | Acupuncture | Self-control | Life quality:① Pain score ②Dysaesthesia ③Walking distance | no | 3 | 3 | no | no | English |
| 2005 | YUAN Yue-bin[192] | Mainland China | Practical Preventive Medicine | Digestive system diseases（Liver disease） | Effect evaluation | Prospective cohort study | Single herbs(Green Tea) | Blank control | Biological indicator:1.ALT，AST;2.PⅢP,Ⅳ-C,HA | no | 715 | 1379 | no | no | Chinese |
| 2005 | Li Ping[193] | Mainland China | Journal of Beijing University of Traditional Chinese Medicine | Respiratory system diseases(SARS) | Effect evaluation | Prospective cohort study | Chinese herbal medicine formulas(unspecified)* | Positive control | the chest x-rays sum | no | 231 | 130 | no | yes | Chinese |
| 2004 | Jonas Persson[194] | Sweden | Psychopharmacology | Others（35-80 years old healthy volunteers） | Effect evaluation | Prospective cohort study | Single herbs(Ginkgo biloba and Ginseng) | Blank control | Memory tests/Neuropsychological/life-style factors | no | Ginkgo biloba: 40; Ginseng:86 | Ginkgo biloba(Blank control): 40; Ginkgo biloba(Positive drug control): 40; Ginseng(Blank control): 86; Ginseng(Positive drug control): 86 | no | yes | English |
| 2004 | NONG Yibing[195] | Mainland China | Chinese Journal of Integrated Chinese and Western Medicine | Circulatory system diseases(acute myocardial infarction) | Prognosis | Retrospective cohort study | Chinese herbal medicine formulas(Unspecified)* | Positive control | Endpoint events | no | 133 | 29 | no | yes | Chinese |
| 2004 | M. Habs[196] | Germany | Forsch Komplementärmed Klass Naturheilkd | Circulatory system diseases(Heart failure) | Economic evaluation+Effect evaluation | Prospective cohort study | Single herbs（Hawthorn Special Extract WS）* | Positive control | Life quality:①Disease-specific (Minnesota Living with Heart Failure Questionnaire) ;②Non-specific (EuroQoL EQ5D) instrument. | no | 588 | 364 | no | no | English |
| 2004 | Patrick Blossfeldt[197] | The United Kingdom | ACUPUNCTURE IN MEDICINE | Motor system diseases(Chronic neck pain) | Effect evaluation | Prospective cohort study | Acupuncture* | Positive control | Life quality:Oral rating scale of improvement presented | no | 172 | no | no | no | English |
| 2004 | LIU Bao-yan[198] | Mainland China | Tianjin Journal of Traditional Chinese Medicine | Respiratory system diseases(SARS) | Effect evaluation | Prospective cohort study | Chinese herbal medicine formulas(unspecified)* | Positive control | the chest x-rays sum | no | 231 | 130 | no | yes | Chinese |
| 2003 | Anthony Stellon[199] | The United Kingdom | ACUPUNCTURE IN MEDICINE | Circulatory system diseases(Valvular heart disease and prosthetic valves) | Adverse events | Prospective cohort study | Acupuncture | No control | The incidence of endocarditis | no | 37 | no | no | no | English |

Reference

[1] ZHOU Yuchen1, et al. Antiviral and antifibrotic therapies reduce occurrence of hepatocellular carcinoma in patients with chronic hepatitis B and liver fibrosis: a 144-week prospective cohort study. J South Med Univ 2019; 39(6):633-640.

[2] FANG Yanyan, et al. Chinese Herbs for Pi Invigorating，Dampness Resolving，Shen Benefiting，and Collaterals Dredging Trea- ted 323 Ankylosing Spondylitis Patients: a Cohort Study. Chinese Journal of Integrated Traditional and Western Medicine 2019; 39(5): 553-556.

[3] LI Lei, et al. Clinical study on treatment of insomnia with nourishing kidney, harmonizing liver and spleen and tranquilizing mind. World Journal of Integrated Traditional and Western Medicine 2019; 14(6): 826-831.

[4] MENG ShuHui, et al. Pharmacoeconomic Evaluation of Getong Tongluo Capsule in the Treatment of Ischemic Stroke. CHINA JOURNAL OF PHARMACEUTICAL ECONOMICS 2019; 14(4): 23-29.

[5] LIU Sutong, et al. Retrospective Cohort Study of Fuzheng Quxie Prescription on Improving Progression-free Survival of Lung Cancer Patients. ACTA CHINESE MEDICINE 2019; 34(254): 1497-1501.

[6] ZHANG Liang, et al. Retrospective cohort study on the effect of Huqi formula on liver cancer with qi deficiency after operation. Journal of Traditional Chinese Medicine 2019; 60(4): 306-310.

[7] Miao Ruiheng，Liu Xiaomeng，Ma Jia, et al. A retrospective cohort study on turtle probing the cave needling at tendon node of neck for treatment of MPS related brachioradialis myalgia. Chinese Journal of health medicine.2019;21(02):104-106.

[8] Jiao Huiduo, Huang Tan, Wu Qunqiang,et al. Cohort Study on Stroke Patients with Upper Limb Dysfunction after Acupuncture Combined with Rehabilitation Training. World Chinese Medicine.2019;14(1):204-208.

[9] Che-Jui, Chang, Yao-Hsu, Pau-Chung Chen, et al. Stomach Cancer and Exposure to Talc Powder without Asbestos via Chinese Herbal Medicine: A Population-Based Cohort Study. International journal of environmental research and public health, 2019;16(717).

[10] Shen T, Liu Y, Shang J, et al. Incidence and Etiology of Drug-Induced Liver Injury in Mainland China. Gastroenterology.2019:1-12.

[11] GONG Zhao-hui，LI Rong，WU Wei，et al. Tongmai Buxin Ointment Combined with Western Medicine in Treating 50 Cases of Acute Myocardial Infarction with Qi Deficiency and Blood Stasis Syndrome: A Retrospective Cohort Study. Journal of Traditional Chinese Medicine.Apr 2019;60(7):576-581. (in Chinese)

[12] CUI Wei-feng，FAN Xiao-hui，WANG Shou-fu，et al. Effect of Long-term Use of Jiangyabao Series of Chinese Patent Medicines on the Outcome of Hypertension：a Cohort Study. Chinese General Practice. Jan 2019; 22(1):101-105. (in Chinese)

[13] Zhang Li-jie, Zhang Gu-cheng, Shen Hong，et al. A cohort study on the reversal of patients with hepatitis B liver cirrhosis during the treatment of integrated Chinese and Western medicine. J Hunan Normal Univ(Med Sci). 2019;16(1):34-37. (in Chinese)

[14] Liang-Yu Chen, Hung-Rong Yen,Mao-Feng Sun，et al.Acupuncture treatment is associated with a decreased risk of developing stroke in patients with depression: A propensity-score matched cohort study. Journal of Affective Disorders.Mar 2019;298-306.https://doi.org/10.1016/j.jad.2019.03.020

[15] Ching-Hui Huang, et al. Traditional Chinese medicine is associated with a decreased risk of heart failure in breast cancer patients receiving doxorubicin treatment. Journal of Ethnopharmacology. doi.org/10.1016/j.jep.2018.09.030

[16] Su-Tso Yang, et al. Utilization of Chinese medicine for respiratory discomforts by patients with a medical history of tuberculosis in Taiwan. BMC Complementary and Alternative Medicine. doi.org/10.1186/s12906-018-2377-4

[17] WEN Jianting, et al. Cohort study on the endpoint events: 323 cases of ankylosing spondylitis. World Journal of Integrated Traditional and Western Medicine 2018; 13(4): 466-470.

[18] FANG Yanyan, et al. The formulas of cohort study of Rheumatoid Arthritis with spleen wet application. Chin J Clin Healthc 2018; 21(4): 505-509.

[19] XU Zhigang, et al. A Retrospective Cohort Study of 30 Cases of Rheumatoid Arthritis with Phlegm and Blood Stasis Treated by Shaogua Xiegong Duan cao Decoction InnerMongolia. Journal of Traditional Chinese Medicine 2018; 37(12): 18-19.

[20] CHEN Menglian, et al. Effect of integrated intervention of traditional Chinese medicine on elderly patients with essential hypertension in community. Herald of Medicine 2018; 37: 13-17.

[21] YUAN Chenhao, et al. Cohort Study on Endpoint Events of Compound Danshen Dripping Pills in Patients with Myocardial Infarction. JOURNAL OF SHANDONG UNIVERSITY OF TCM 2018; 42(5): 414-417.

[22] Wen Jianting, et al. Cohort study on the endpoint events in 1904 patients with osteoarthritis. Chin J Clin Healthc 2018; 21(4): 456-461.

[23] Lin Ruizhu, XU Jianfeng, MA Chuan,et al. Retrospective cohort study on the effect of double moxibustion combined with intermittent catheterization on urination diary of neurogenic bladder patients after spinal cord injury. West China Medical Journal.2018;33(10):1272-1276.

[24] LUO Xiaozhou，TANG Chunzhi. A Prospective Cohort Study of the Treatment of Periarteritis of Shoulder with The Conduction Effect of Zhongzhu Acupoints Based on The Propensity Score Method. Journal of Basic Chinese Medicine.2018;24(4):523-526.

[25] ZHU Dao-cheng，LENG Cheng，XIONG Jun et al.Thermosensitive Moxibustion Induces a Better Therapeutic Effect in the Treatment of Facial Paralysis Patients. Acupuncture Research.2018;43(10):666-669.

[26] ZHANG Ye, YANG Xin-Jing, WANG Si-Han, et al. Clinical Study of Electro-acupuncture and Antidepressants on Depression Based on Real World Study Principle. Journal of Basic Chinese Medicine.2018;24(3):374-377.

[27] Zhixin Y, Jianliang Y. Qi end, Shixuan bloodletting combined with Thunder Fire moxibustion in the treatment of Peripheral nerve injury after chemotherapy: a report of 28 cases. Journal of External Therapy of TCM.2018;27(5):31-32.

[28] Zhang Wei, Wu Mingdan, Yang Yi, et al.Effect of Acupuncture Combied with Motor Relearning Program on Upper Limb Function in Patients with Ischemic Stroke: A Presprctive Cohort Study. Journal of Clinical Acupuncture and Moxibustion.2018;34(9):36-39.

[29] GAO Wu-lin，DAI Guo-hua，SHI Xiao-jing, et al. Efficacy of Traditional Chinese Medicine in Treatment of Unstable Angina. Chinese Journal of Experimental Traditional Medical Formulae.2018;24(7):228-234.

[30] GAO Wu-lin，DAI Guo-hua，WU Bin et al. Cohort Study on Effect of Traditional Chinese Medicine in Treating Patients with Myocardial Infarction Complicated with Hypertension. Li Shi Zhen Medicine and Materia Medica Research.2018;29(9):2287-2290.

[31] GAO Wu-lin, DAI Guo-hua, WU Bin et al. Cohort study on intervention effects of traditional Chinese medicine on the combination disease of myocardial infarction and hyperlipidemia. China Journal of Traditional Chinese Medicine and Pharmacy.2018;33(5):2002-2006.

[32] YUAN Jun，JIN Yan-tao, JIANG Zi-qiang, et al. Retrospective Analysis of the Effect of Chinese Medicine Treatment on CD4+Lymphocyte Count of HIV/AIDS Patients. Chinese Journal of Integrated Traditional and Western Medicine.2018;38(4):407-409.

[33] CHEN Yang，WANG Ming- quan，SONG Wen，et al. Retrospective cohort study on the effect of comprehensive treatment of Chinese medicine on the survival of patients with advanced hepatocellular carcinoma. Journal of Hepatobiliary Surgery.2018;26(4):292-296.

[34] Simma I, Simma L, Fleckenstein J. Muscular diagnostics and the feasibility of microsystem acupuncture as a potential adjunct in the treatment of painful temporomandibular disorders: results of a retrospective cohort study. Acupuncture in Medicine. 2018.

[35] DONG Wen-zhe,LIU Jian,WAN Lei,et al. A Cohort Study on the Occurrence of End-point Events during the Follow-up Period in Patients with Rheumatoid Arthritis Treated with Chinese Medicine.Rheumatism and Arthritis. Mar 2018;7(3):18-22. (in Chinese)

[36] Li Xin,Li Ni,Wang Gang,et al.Tea consumption and the risk of lung cancer in Chinese males: a prospective cohort study. Chin J Prev Med.May 2018;52(5):511-516.（in Chinese）

[37] ZHENG Shi-jing，LI Xue，NIE Chun-li，et al. Study on the Effectiveness and Safety of Lyu Renhe's Syndrome Differentiation of Renal Collateral Syndrome by Queue Strategy. WORLD CHINESE MEDICINE.Jun 2018; 13(6):1342-1346. (in Chinese)

[38] TIAN Zhong-hua，DONG Yong-shu，CUI Wei-feng，et al. Prospective Cohort Study on reducing the recurrence rate of stroke of making use of ZhongFeng Capsule. LISHIZHEN MEDICINE AND MATERIA MEDICA RESAERCH.2018; 29(8):1894-1896. (in Chinese)

[39] DENG Peng，HU Dan，WU Jian-guang，et al. Chinese Medicine Hot Compress Therapy for Treatment of 50 Chronic Stable Angina Pectoris Patients with Pattern of Yin Cold Congelation and Stagnation: A Retrospective Cohort Study. Journal of Traditional Chinese Medicine.Mar 2018;59(5):398-401. (in Chinese)

[40] Mei-Yao Wu,Ming-Cheng Huang,Hou-Hsun Liao，et al.Acupuncture decreased the risk of coronary heart disease in patients with rheumatoid arthritis in Taiwan: a Nationwide propensity score-matched study. BMC Complementary and Alternative Medicine.2018;18:341.https://doi.org/10.1186/s12906-018-2384-5

[41] Yi-Ting Kuo, et al. Complementary Chinese Herbal Medicine Therapy Improves Survival of Patients with Pancreatic Cancer in Taiwan A Nationwide Population-Based Cohort Study. Integrative Cancer Therapies 2018, Vol. 17(2): 411-422.

[42] AIHEMAITI.Abudureyimu, et al. Effect of Taiqipeiyuan Granules on TLR-4 Receptor of HIV-infected Patients Who were Deficency of both Qi-yin and Lung-kidney. Chinese Journal of Medicinal Guide 2017; 19(12): 1373-1377."

[43] WEI Yeye, et al. Observation of Clinical Curative Effect of Taiqipeiyuan Granules to HIV-infected Patients. Chinese Journal of Medicinal Guide 2017; 19(12): 1378-1381.

[44] MENG Yuan, et al. A cohort study on treatment of chronic kidney disease 3 with qi deficiency and dampness⁃heat type by qingbudigui decoction. The Journal of Practical Medicine 2017; 33(20): 3472-3476.

[45] WEN Jianting, et al. A cohort study of endpoint events in 1468 patients with rheumatoid arthritis. J ANHUI UNIV CHINESE MED 2017; 36(5): 13-17.

[46] ZHU Haitao, et al. Curative Effect of Standard Treatment Combined with Rhubarb Enema in Treating Mild Acute Pancreatitis. JOURNAL OF GUIZHOU MEDICAL UNIVERSITY 2017; 42(11): 1311-1314.

[47] JIANG Fangchao, et al. Bushen Jiangzhuo decoction in the treatment of senile hypertension patients of yin and yang deficiency. Jilin Journal of Chinese Medicine 2017; 37(9): 911-915.

[48] FANG Yanyan, et al. Cohort Study of 1658 Cases of Osteoarthritis Treated with Formula of Fortifying the Spleen and Removing Dampness,Tonifying Kidney and Dredging Collaterals. Rheumatism and Arthritis 2017; 6(8): 15-19.

[49] WANG Haibing, et al. Pharmacoeconomic Evaluation of Prospective Observation of Soft Blood Tissue Injury Treated by Huoxue Zhitong Soft Capsule. Chinese Journal of Drug Evaluation 2017; 34(4): 309-313.

[50] TAO Li, et al. Bidirectional cohort study on the influence of syndrome differentiation treatment of Weichang’an Prescription on prognosis of unresectable colorectal liver metastases based on survival analysis. Shanghai Journal of Traditional Chinese Medicine 2017; 51(1): 42-49.

[51] Haiyan Xu , Jun X , Hong Fu. Difference Moxibustion Sensation and Moxibustion Effect in the Treatment of Periarthritis of Shoulder Based on Propensity Score: A Prospective Cohort Study. Liaoning Journal of Traditional Chinese Medicine.2017;44(1):137-140.

[52] Xu Wei, Chen rixin. A prospective cohort study on the effect of heat-sensitive moxibustion on knee osteoarthritis with different moxibustion sensation. Jiang Xi Journal of Traditional Chinese Medicine.2017;48(414):52-55.

[53] Wang Li, Li Jing, Gui Jingui, et al. Clinical Effect of Acupoint Sticking in Preventing and Treating Bronchial Asthma in Children: A Cohort Study. Journal of Anhui University of Chinese Medicine.2017;36(4):44-46.

[54] Wang Jianbin, Yang Yufei, Han Zhiyu, et al. A Retrospective Cohort Study of Traditional Chinese Medicine on Primary Liver Cancer after Percutaneous Microwave Ablation. Journal of Medical Research.2017;46(1):49-52.

[55] XUE Mei－ping, LIU Li－kun, GUO Yan－rong,et al. Clinical Study on Small Cell Lung Cancer in the Maintenance Treatment with Chinese Herbal medicine. World Journal of Integrated Traditional and Western Medicine.2017;12(9):1271-1274.

[56] Yin-Yin Lin，I-Yun Lee，Wen-Shih Huang，et al. Danshen improves survival of patients with colon cancer and dihydroisotanshinone I inhibit the proliferation of colon cancer cells via apoptosis and skp2 signaling pathway. Journal of Ethnopharmacology.2017

[57] Tsai-Hui Lin, Hung-Rong Yen, Jen-Huai Chiang,et al. The use of Chinese herbal medicine as an adjuvant therapy to reduce incidence of chronic hepatitis in colon cancer patients: A Taiwanese population-based cohort study. Journal of Ethnopharmacology.2017;202(2017):225-233.

[58] Qi Shi, Shanshan Liu, Wen Li, et al. Exploring the medication duration based on the effect of traditional Chinese medicine on postoperative stage I-III colorectal patients: a retrospective cohort study. Oncotarget.2017;8(8): 13488-13495

[59] Kuo-Chin Huang, Hung-Rong Yen, Jen-Huai Chiang, et al. Chinese Herbal Medicine as an Adjunctive Therapy Ameliorated the Incidence of Chronic Hepatitis in Patient with Breast Cancer: A Nationwide Population-Based Cohort Study. Evidence-Based Complementary and Alternative Medicine.2017:9

[60] Jiang Zhi-yan，Wang Xue-feng，Wang Li-ning，et al.Multi-Center Efficacy Evaluation Study of Treating Pediatric Mycoplasma Pneumonia with Chinese Medicine Integrated with Western Medicine. WORLD CHINESE MEDICINE Mar 2017;12(3):536-539. (in Chinese)

[61] WANG Pei-yu, ZHAO Jun-zhao, LIN Jia，et al.Comparison of the effect of traditional Chinese medicine and Voluven on preventing ovarian hyper-stimulation syndrome during in vitro fertilization and embryo transfer. Journal of Wenzhou Medical University. Feb 2017;47(2):104-109. (in Chinese)

[62] DONG Ya-nan，NI Sai-sai，DAI Li，et al.The Impact of the TCM Syndrome Differentiation Treatment Combined with Antiviral Treatment on the Incidence of HBV Related Decompensated Liver Cirrhosis.Journal of Yunnan University of Traditional Chinese Medicine.Jun 2017;40(3):41-46. (in Chinese)

[63] Kuo-Feng Hung,Ching-Ping Hsu,Jen-Huai Chiang，et al. Complementary Chinese Herbal Medicine Therapy Improves Survival of Patients with Gastric Cancer in Taiwan: A Nationwide Retrospective Matched-Cohort Study. Journal of Ethnopharmacology.2017.http://dx.doi.org/10.1016/j.jep.2017.02.004

[64] Fuu-Jen Tsai, Tsung-Jung Ho, Chi-Fung Cheng，et al. Effect of Chinese herbal medicine on stroke patients with type 2 diabetes. Journal of Ethnopharmacology.2017.http://dx.doi.org/10.1016/j.jep.2017.02.024

[65] Kuen-Hau Chen, Ming-Hsien Yeh, Hanoch Livneh，et al. Association of traditional Chinese medicine therapy and the risk of dementia in patients with hypertension: a nationwide population-based cohort study.BMC Complementary and Alternative Medicine.2017;17:178. DOI 10.1186/s12906-017-1677-4

[66] Shun-Ku Lin, Po-Hung Lin, Ren-Jun Hsu，et al.Traditional Chinese Medicine Therapy Reduces the Catheter Indwelling Risk in Dementia Patients with Difficult Voiding Symptoms. Journal of Ethnopharmacology.2017.http://dx.doi.org/10.1016/j.jep.2017.03.040

[67] Mei-Yao Wu, Ming-Cheng Huang,Jen-Huai Chiang，et al.Acupuncture decreased the risk of coronary heart disease in patients with fibromyalgia in Taiwan: a nationwide matched cohort study.Arthritis Research & Therapy.2017;19:37.DOI 10.1186/s13075-017-1239-7

[68] ZHANG Tong, et al.Cohort Study on Prognosis of Patients with Metastatic Colorectal Cancer Treated with Integrated Chinese and Western Medicine. Chinese Journal of Integrative Medicine 2017; 1-6.

[69] Yu-Jun Wang, et al. The Effectiveness of Traditional Chinese Medicine in Treating Patients with Leukemia. Evidence-Based Complementary and Alternative Medicine. Volume 2016, Article ID 8394850, 12 pages

[70] Yu-Chiang Hung, et al. Adjuvant Chinese Herbal Products for Preventing Ischemic Stroke in Patients with Atrial Fibrillation. PLOS ONE 2016; 11(7): e0159333.

[71] Tzung-Yi Tsai, et al. Decreased risk of stroke in patients receiving Traditional Chinese Medicine for vertigo: a population-based cohort study. Journal of Ethnopharmacology 2016; dx.doi.org/10.1016/j.jep.2016.03.008

[72] Yuchao L, Juntao Y, Zhenyu W, et al. Effect of Yijin Meridian on skeletal muscle contractile function in elderly patients with skeletal muscle decrease. Academic Journal of Shanghai University of Traditional Chinese Medicine. 2016;30(5):42-45.

[73] Jun X, Lin J, Ding-Yi X, et al. Effects of heat-sensitive moxibustion in the treatment of knee osteoarthritis(swelling phase) based on propensity acore: A prospective cohort study. China Journal of Traditional Chinese Medicine and Pharmacy.2016;31(6):2295-2298.

"[74] San-Yuan Wu, Huey-Yi Chen, Kao-Sung Tsai, et al. Long-Term Therapy With Wu-Ling-San, a Popular Antilithic Chinese Herbal Formula, Did Not Prevent Subsequent Stone Surgery:

A Nationwide Population-Based Cohort Study. The Journal of Health Care Organization, Provision, and Financing.2016; 53: 1–7."

[75] Min Dai, Yue-Wu Yang, Wen-Hai Guo, et al. Addition and Subtraction Theory of TCM Using Xiao-Chaihu-Decoction and Naturopathy in Predicting Survival Outcomes of Primary Liver Cancer Patients: A Prospective Cohort Study.2016：9

[76] Kiguba R, Ononge S, Karamagi C, et al. Herbal medicine use and linked suspected adverse drug reactions in a prospective cohort of Ugandan inpatients. BMC Complementary and Alternative Medicine.2016;16(1):145-152.

[77] HUANG He,CAI Li-qun,CHI Wei，et al. Application of pull-thread and medicated-thread therapy in the treatment of posterior horseshoe-shaped perianal abscess：A prospective cohort study. CHINA MODERN DOCTOR.Jul 2016;54(21):46-49. (in Chinese)

[78] Liu Zhong-liang, Li Fei-ze，Zhou guo-er，et al. Observation of Yangfei Xiaoji Decotion in the treatment of advanced non-small cell lung cancer.Zhejiang JITCWM.2016;26(6):542-545. (in Chinese)

[79] ZHANG Ying，YANG Wei，JIANG Junjie，et al. Impact Analysis of the Clinical Use of Shenfu Injection on Liver Function by Using Propensity Score Methods.JOURNAL OF LIAONING UNIVERSITY OF TCM.Sep 2016;18(9):66-70. (in Chinese)

[80] Wang Jian-bin, Yang Yu-fei, Wu Yu，et al.A Cohort Study of Traditional Chinese Medicine (TCM) on Primary Liver Cancer in the Recurrence and Metastasis after Microwave Ablation.World Science and Technology modernization of Traditional Chinese Medicine and Materia Medica.2016;18(10):1640-1645. (in Chinese)

[81] Tzung-Yi Tsai, Chung-Yi Li, Hanoch Livneh，et al. Decreased risk of stroke in patients receiving Traditional Chinese Medicine for vertigo: a population-based cohort study. Journal of Ethnopharmacology.2016. http://dx.doi.org/10.1016/j.jep.2016.03.008

[82] Tom Fleischer,Tung-Ti Chang,Jen-Huai Chiang,et al.Improved Survival With Integration of Chinese Herbal Medicine Therapy in Patients With Acute Myeloid Leukemia: A Nationwide Population-Based Cohort Study. Integrative Cancer Therapies.Aug 2016.DOI: 10.1177/1534735416664171

[83] Jui-Ming, Liu,Po-Hung Lin,Ren-Jun Hsu, et al. Complementary traditional Chinese medicine therapy improves survival in patients with metastatic prostate cancer.Medicine.2016;95:31.http://dx.doi.org/10.1097/MD.0000000000004475

[84] Danielle M. Graff,Mark J. McDonald.Auricular Acupuncture for the Treatment of Pediatric Migraines in the Emergency Department.Pediatric Emergency Care.2016.

[85] Yantao Jin, et al. Survival of AIDS patients treated with traditional Chinese medicine in rural central China a retrospective cohort study. Evidence-Based Complementary and Alternative Medicine. Volume 2015, Article ID 282819, 7 pages

[86] Shanshan Liu, et al. Comparison of tripterygium wilfordii multiglycosides and tacrolimus in the treatment of idiopathic membranous nephropathy a prospective cohort study. BMC Nephrology 2015; 16:200-208.

[87] Der-Shiang Tsai, et al. The use of Chinese herbal medicines associated with reduced mortality in chronic hepatitis B patients receiving lamivudine treatment. .Journal of Ethnopharmacology 2015; 174:161-167.

[88] Ya Yuwen, et al. The add-on effect of a Chinese herbal formula for patients with resistant hypertension: study protocol for a pilot cohort study. Journal of Integrative Medicine 2015; 13(2): 122-128.

[89] Hsing-Yu Chen, et al. Use of traditional Chinese medicine reduces exposure to corticosteroid among atopic dermatitis children_ A 1-year follow-up cohort study. Journal of Ethnopharmacology 2015; 159: 189-196.

[90] TANG Zhen-qing, et al. Efficacy comparison of treating anxiety symptom of community rehabilitation personnel with Jitai Tablets and methadone. China Journal of Traditional Chinese Medicine and Pharmacy 2015; 30(2): 613-616.

[91] Dai Guohua, et al. A cohort study of 238 patients with myocardial infarction with endpoint events. Journal of Traditional Chinese Medicine 2015; 56(1): 31-35.

[92] DAI Guohua, et al. Cohort Study of the Application of the Formulas for Nourishing Yin Reducing Fire and Calming Down the Mind in 313 Cases of Arrhythmia in the Patients of Coronary Heart Disease. World Journal of Integrated Traditional and Western Medicine 2015; 10(3): 403-406.

[93] ZHENG Wenguang, et al. Cohort study of outcome event of traditional Chinese medicine on coronary heart disease patients with arrhythmia during follow-up Hebei. Journal of Traditional Chinese Medicine 2015; 37(5): 669-672.

[94] WU Maolin, et al. Evaluation of Clinical Effects of Modified BanXia XieXin Tang in Treating Advanced Esophageal Cancer Western. Journal of Traditional Chinese Medicine 2015; 28(5): 50-52.

[95] Meili Y, Xiaomei H, Hao X. Clinical Evaluation on Dog-days Paste Treating Winter Diseases in Summer for the Prevention and Treatment of Angina Pectoris. Chinese Journal of Integrative Medicine on Cardio-/Cerebrovascular Disease.2015;13(9):1086-1089.

[96] Jun X, Lele G, Zhenhai C, et al. Observation on the Therapeutic effect of moxibustion on 60 cases of Acute Lumbar Intervertebral Disc Herniation with different moxibustion sensation. Journal of Traditional Chinese Medicine. 2015, 56(21):1836-1839.

[97] Jingru L, Jianping M, Xiulan M, et al. Clinical observation of moxibustion in the treatment of AIDS diarrhea with spleen and stomach weakness .World Science and Technology/Modernization of Traditional Chinese Medicine and Materia Medica.2016;18(2):237-240.

[98] Zhenping L, Wenyu L, Lihua F. Clinical study of electroacupuncture on 415 cases with hyperplasia of mammary glands. Journal of Shanxi College of Traditional Chinese Medicine.2015;16(3):61-62

[99] Xiong Jun, Zhang Wei, Jiao Lin, et al. Different Warm Sensations May Induce Different Therapeutic Effects in Primary Dysmenorrhea Patients Undergoing Moxibustion Based on Propensity Score: A Prospective Cohort Study. Acupuncture Research.2015;40(6):465-469.

[100] FAN Junming, QIAO Shan, LIU Peng, et al. Retrospective analysis on acupuncture in treatment of cerebral infarction evaluated with propensity score. Chinese Acupuncture & Moxibustion.2015;35(1):72-76.

[101] Rui Liu, Shu lin He, Yuan chen Zhao, et al. Chinese Herbal Decoction Based on Syndrome Differentiation as Maintenance Therapy in Patients with Extensive-Stage Small-Cell Lung Cancer: An Exploratory and Small Prospective Cohort Study. Evidence-Based Complementary and Alternative Medicine.2015:12.

[102] Chih-Wen Chiu1, Tsung-Chieh Lee2, Po-Chi Hsu ,et al. Efficacy and safety of acupuncture for dizziness and vertigo in emergency department: a pilot cohort study. BMC Complementary and Alternative Medicine.2015;15(1):173.

[103] MAO Jun，CUI Liu-fu，WU Shou-ling，et al. Effects of tea consumption on the risk of cerebral infarction. Chin J of Clinical Rational Drug Use.Jan 2015;8(1):15-16.（in Chinese）

[104] SHI Lin，FU Qi，XU Wei-ru，et al. Clinical evaluation on traditional Chinese medicine treatment for breast cancer patients with high risks of recurrence and metastasis. Chinese Clinical Oncology.Oct 2015; 20(10):885-889. (in Chinese)

[105] Fabian aus dem siepen,ralf Bauer, Matthias aurich，et al. Green tea extract as a treatment for patients with wild-type transthyretin amyloidosis: an observational study. Drug Design, Development and Therapy.Dec 2015;9:6319-6325.

[106] Hsienhsueh Elley Chiu1, et al. Favorable circulatory system outcomes as adjuvant traditional Chinese medicine (TCM) treatment for cerebrovascular diseases in Taiwan. PLOS ONE January 2014; 9(1): 1-6.

[107] Yantao Jin, et al. Traditional Chinese medicine could increase the survival of people living with HIV in rural central China_ a retrospective cohort study, 2004-2012. The American Journal of Chinese Medicine 2014; 42(6):1333–1344.

[108] Wei Zhang, et al. Effect of cantharidins in chemotherapy for hepatoma_ a retrospective cohort study. The American Journal of Chinese Medicine 2014; 42(3):561-567.

[109] Hai-Lu Zhao, et al. Eight-year survival of AIDS patients treated with Chinese herbal medicine. The American Journal of Chinese Medicine 2014; 42(2): 261-274.

[110] Yuan-Wen Lee, et al. Adjunctive traditional Chinese medicine therapy improves survival in patients with advanced breast cancer a population-based study. Cancer DOI: 10.1002/cncr.28579.

[111] Zhu-Qing Ji, et al. Safety of Brucea javanica and cantharidin combined with chemotherapy for treatment of NSCLC patients. Journal of Integrative Medicine 2014;15(20): 8603-8605.

[112] Yu-Chiang Hung, et al. Integrated traditional Chinese medicine for childhood asthma in Taiwan a Nationwide cohort study. Complementary and Alternative Medicine 2014; 14: 389-395.

[113] ZHU Huayu, et al. A retrospective cohort study of traditional Chinese medicine treatment and surgical treatment for granulomatous mastitis. LISHIZHEN MEDICINE AND MATERIA MEDICA RESEARCH 2014; 25(3): 635-637.

[114] WANG Zhiying, et al. Therapeutic Effect and Mechanism on Tonifying Lung and Kidney and Eliminating Wind and Resolving Phlegm Treating Chronic Persistent Asthma. Journal of Nanjin University of TCM 2014; 30(4): 316-319.

[115] XU Danping, et al. Treatment of patients with coronary heart disease with Deng Tietao coronary heart disease. Chinese Journal of Gerontology 2014; 34: 1167-1168.

[116] QIU Yiwen, et al. Effect of traditional Chinese medicine on survival of advanced primary liver cancer: Multicenter retrospective cohort study. Journal of Guangzhou University of Traditional Chinese Medicine 2014; 31(5): 699-705.

[117] SUN Zhen, et al. Traditional Chinese medicine in improving overall survival rates for small hepatocellular carcinoma after resection: a retrospective cohort study. JOURNAL OF LIAONING UNIVERSITY OF TCM 2014; 16(12): 63-66.

[118] Shih C C, Hsu Y T, Wang H H, et al. Decreased Risk of Stroke in Patients with Traumatic Brain Injury Receiving Acupuncture Treatment: A Population-Based Retrospective Cohort Study. PLoS ONE.2014;9(2).

[119] Shu Peng, Liu Shen-lin，Wang Rui-ping，et al. A clinical study of 201 cases of gastric cancer recurrence and metastasis treated by Yiqi Huayu Jiedu Decoction. Jiangsu Journal of Traditional Chinese Medicine.2014 ;46(4):23-24. (in Chinese)

[120] Zhou Dai-han，Lin Li-zhu，Tian Hua-qin，et al. Effect on the survival of elderly patients with non-small cell lung cancer treated by the traditional Chinese medicine treatment based on Yiqi Huatan method：a multicenter，clinically prospective cohort study.WORLD CHINESE MEDICINE.Jul 2014;9(7):833-838. (in Chinese)

[121] Shuo-Meng Wang,Ming-Nan Lai, Alan Wei，et al. Increased Risk of Urinary Tract Cancer in ESRD Patients Associated with Usage of Chinese Herbal Products Suspected of Containing Aristolochic Acid. PLOS ONE.Aug 2014; 9(8).

[122] Youfu Ke, et al. Essential hypertension treated by wuling powder and modified tianma gouteng decoction_ a cohort study without controls. Complementary Therapies in Medicine 2013; 21:609-612.

[123] Yueh-Hsiang Huang, et al. Influence of Chinese medicine on weight loss and quality of life during radiotherapy in head and neck cancer. Integrative Cancer Therapies 2013; 12(1): 41-49.

[124] WANG Jing, et al. Clinical Efficacy Qi-Clearing Construction-Cooling Decoction on Severe Sepsis: A Clinical observation of 30 cases Guiding. Journal of Traditional Chinese Medicine and Pharmacy 2013; 19(6): 16-18.

[125] LIU Guoqing, et al. Curative effect study on the treatment of middle and lower tibia fracture with intramedullary nail combined with Taohong Siwu Jiegu formula. Chinese Journal of Health Care Nutrition 2013; 9: 817-818.

[126] ZHU Weirong, et al. Effect of Chinese Medicine Fuzheng Quxie Regimen on the survival status of patients with pancreatic cancer: a cohort study. Chin J Integr Trad West Med Dig 2013; 21(12): 632-635.

[127] QIU Ruijin, et al. Effect of Shugan Yishen formula on quality of life of patients with HER-2 positive breast cancer. Journal of Changchun University of Traditional Chinese Medicine 2013; 29(4): 587-589.

[128] Cong Z, Yan L, Yu Z, et al. A cohort study on effect of exercise of baduanjin on TCM symptoms in people with fatigue-predominant sub-health. Journal of Beijing University of Traditional Chinese Medicine · Clinical Medicine.2013;20(6):6-9

[129] Yin Dongfeng，Gao Hong，Zhou Wenbo. Clinical research of the compliance of endocrinotherapy for advanced breast cancer patients with TCM therapy. Modern Oncology.2013;21(5):1050-1053.

[130] Ochi, James W. Acupuncture instead of codeine for tonsillectomy pain in children. International Journal of Pediatric Otorhinolaryngology.2013; 77(12):2058-2062.

[131] Choi J S, Han J Y, Ahn H K, et al. Fetal and Neonatal Outcomes in Women Reporting Ingestion of Licorice (Glycyrrhiza uralensis) during Pregnancy. Planta Medica.2013;79:97-101.

[132] ZHOU Yu-qian，MAO Wen-qin，ZHANG Xiao-jun, et al. Effects of　Shugan　Jianpi　Gusui　Recipe on Multiple Sclerosis Recurrence:a Primary Report. CJITWM.Jan 2013;33(1):31-34. (in Chinese)

[133] HUANG Qi.A retrospective study on the treatment of coronary heart disease ventricular premature beat by nourishing blood and relieving wind.JETCM.Aug 2013;22(8):1321-1322. (in Chinese)

[134] Zhu Dong, Shi Qi，Wang Yong-jun，et al. Ambispective Cohort Study on Treatment of Cervical Spondylotic Myelopathy with Qi-blood Regulation Method. World Science and Technology modernization of Traditional Chinese Medicine and Materia Medica.2013;15(5):1067-1073. (in Chinese)

[135] ZHAO Yuan.Prospective cohort study on rehabilitation therapy of knee degenerative osteoarthritis with integrated Chinese traditional and western medicine. Practical Journal of Clinical Medicine.Jan 2013;10(1):138-140. (in Chinese)

[136] ZHAO Xiao-feng, et al.Mortality and recurrence of vascular disease among stroke patients treated with combined TCM therapy. Journal of Traditional Chinese Medicine 2012 June 15; 32(2): 173-178.

[137] DUAN Wen-hui, LU Fang, LI Li-zhi, et al. Clinical Efficacy of Traditional Chinese Medicine on Acute Myocardial Infarction——A Prospective Cohort Study. Chin J Integr Med 2012 Nov; 18(11):807-821.

[138] Hu Xiaoyu, et al. A prospective cohort study on the influence of high doses of herbs for clearing heat and resolving stasis on survival rates in patients with hepatitis B-related acute on chronic liver failure. Journal of Chinese Integrative Medicine 2012; 10(2):176-185 .

[139] ZHANG Tao, et al. Effects of Entecavlr and Shenxian Yiganling Combination Therapy on patients with HBeAg-positive Chronic Hepatitis B for 48 Weeks. Chinese Journal of Integrated Traditional and Western Medicine 2012; 32(2): 180-182.

[140] ZHANG Liling, et al. Clinical observation on the treatment of common Hand-foot-and-mouth disease with traditional Chinese medicine. Nei Mongol Journal of Traditional Chinese Medicine 2010; DOI:10.16040/j.cnki.cn15-1101.2012.16.023.

[141] ZHANG Guoliang, et al. Clinical observation on Different Therapies for 837 Infants with Popular Type of Hand-Foot-Mouth Disease. Journal of Traditional Chinese Medicine 2012; 53(5): 395-402.

[142] WANG Zhiying, et al. Clinical Study on Preventive Treatment of Tonifying Lung and Kidney, Eliminating Wind and Resolving Phlegm for Recurrence of Asthma. Journal of Nanjin University of TCM 2012; 28(5): 421-424.

[143] WAN Chanjun, et al. Retrospective cohort study on treatment of cardiac x syndrome with Liqi Kuanxiong Huoxue circulation. Beiiing Journal of Traditional Chinese Medicine 2012; 31(3): 175-178.

[144] White A, Richardson M, Richmond P, et al. Group acupuncture for knee pain: evaluation of a cost-saving initiative in the health service. Acupuncture in Medicine.2012;30(3):170-175.

[145] GAO Hui，WANG Tao，YU Ren-huan, et al. A Cohort Study on Delaying the Progress of Chronic Renal Failure Mainly with Modified Shenqi Dihuan Decoction. CJITWM.Jan 2012;32(1):39-42. (in Chinese)

[146] CHEN Xiao-zhong，CHEN Bing，WU Yu-nan, et al. Clinical Observation of Integrated Chinese and Western Medines on 68 Cases of Chronic Hepatitis.HUNAN JOURNAL OF TRADITIONAL CHINESE MEDICINE.Mar 2012;28(2):7-9. (in Chinese)

[147] LI Qing，ZHANG Hui-min，FEI Yu-tong, et al.Treatment of Diabetic Nephropathy by Integrative Medicine：a Multi-center Prospective Cohort Study. CJITWM.Mar 2012;32(3):317-321. (in Chinese)

[148] Julia A.Montague,Lesley M.Butler,Anna H.Wu.Increased Risk of Urinary Tract Cancer in ESRD Patients Associated with Usage of Chinese Herbal Products Suspected of Containing Aristolochic Acid. Cancer Causes Control.Aug 2012;23:1635-1641.

[149] LIU Qingchi, et al. Clinical Study on Treatment of Shenyangxu of Chronic Aplastic Anemia with Longteng Shengxue Pill. Chinese Journal of Experimental Traditional Medical Formulae 2011; 17(18): 256-259.

[150] GUAN Ruodan, et al. A retrospective cohort study on the prevention and treatment of short-term recurrence and metastasis of breast cancer by Huaier Granule. Guangdong Medical Journal 2011; 32(11): 1490-1492.

[151] LIU Jing, et al. Observation of Jianpi Compoud Recipe combined with chemotherapy in post-operation colorectal cancer patients. Journal of Clinical Medicine in Practice 2011; 15(7): 29-32.

[152] LU Wenping, et al. A prospective cohort study on hormone-dependant metastatic breast cancer treated with Shuganyishen formula. International Journal of Traditional Chinese Medicine 2011; 33(5): 389-393.

[153] Yan L, Yin L, Cong Z, et al. Improvement effect of practicing Baduanjin on life quality in people group of fatigue-predominant sub-health. Journal of Beijing University of Traditional Chinese Medicine.2011;34(3):209-212.

[154] Jin gang D, Cong Z, Yan L, et al. A cohort study on the effect of training Baduanjin on the improvement of basic physical fitness of fatigue sub-healthy people. Chinese Journal of Basic Medicine in Traditional Chinese Medicine .2011;(02):76-78.

[155] Jianling M, Xiaowei G. Clinical Nursing observation of balance Acupuncture in the treatment of Hemodialysis Hypertension. Modern Journal of Integrated Traditional Chinese and Western Medicine.2011;20(29):3741-3742.

[156] ZHONG Run-fen, HUANG Shi-xi. Observation on therapeutic effect of acupuncture on spontaneous facial paralysis in acute stage. Chinese Acupuncture & Moxibustion.2011;31(7):587-590.

[157] Michael McCulloch, Michael Broffman, Mark van der Laan, et al. Colon Cancer Survival With Herbal Medicine and Vitamins Combined With Standard Therapy in a Whole-Systems Approach: Ten-Year Follow-up Data Analyzed With Marginal Structural Models and Propensity Score Methods. Integrative Cancer Therapies.2011;10(3):240–259.

[158] YANG Chang-kun,CHEN Jian-jie.A 5-year retrospective study on the treatment of chronic hepatitis b by TCM syndrome differentiation. Journal of Changchun University of Traditional Chinese Medicine.Apr 2011;27(2):241-242. (in Chinese)

[159] Pei-Jen Chang, et al. Use of Sheng-Hua-Tang and health-related quality of life in postpartum women a population-based cohort study in Taiwan.International Journal of Nursing Studies 2010; 47:13-19.

[160] ZHANG Guoliang, et al. Clinical efficacy of 334 patients with common Hand-foot-and-mouth disease. Anhui Medical Journal 2010; 31(12): 1412-1414.

[161] LIU Zhaoyang, et al. Syndrome Types and Treatment According to Syndrome Differentiation in Women with Convalescent Cerebral Concussion:a Prospective Cohort Study. Chinese J T rad Med T raum & Orthop 2010; 18(5): 41-43.

[162] HUANG Wei, et al. Retrospective Cohort Study on Clinical Value of Huaier Granule in Postoperative Patients with Liver Transplantation for Hepatocellular Carcinoma. Chin J Bases Clin General Surg 2010; 17(6): 547-551.

[163] Xiaoping Z, Ding L, Bin Y, Comparison of immediate effects of scraping, cupping and electroacupuncture in the treatment of cold and headache. Li Shi Zhen Medicine and Materia Medica Research.2010;21(7):1827-1828

[164] Fan Yuling, Jiang Xiangjun, et al. Clinical observation of Acupuncture and moxibustion at back Shu Point combined with Zhisou Powder in the treatment of cough after infection. Medical Information.2010;9:2626-2627.

[165] WU Shi-yan, CHEN Yue, JIANG Ting-hui, et al. Study on the clinical effictiveness of Ling Yaoxing in the treatment of liver metastasis of colorectal cancer by Queue Strategy. Journal of Traditional Chinese Medicine.2010;3:38-41. (in Chinese)

[166] ZHU Hui, LUO Xing-ping, WANG Li-jie, et al. Evaluation on Clinical Effect of Long-term ShexiangBaoxin Pill Administration for Treatment of Coronary Heart Disease. CJITWM.May 2010;30(5):474-477. (in Chinese)

[167] GUAN Jia-hui,YANG Yu-fei,WU Yu, et al. Combine traditional Chinese and western medicine treatment to reduce phase II-III the post-operation relapse and metastasis, 222 cases of colorectal cancer effect a radical cure to follow-up cohort study. ONCOLOGY PROGRESS.Mar 2010;8(2):193-195. (in Chinese)

[168] Sang-Wook Yi, et al. Association between ginseng intake and mortality_ Kangwha cohort study. THE JOURNAL OF ALTERNATIVE AND COMPLEMENTARY MEDICINE 2009; 15(8): 921–928.

[169] CHEN Jian, et al. A retrospective cohort study on the treatment of Hand-foot-and-mouth disease in children with damp and heat. China Journal of Traditional Chinese Medicine and Pharmacy 2009; 1: 19-20.

[170] Simcock R, Fallowfield L, Jenkins V. Group acupuncture to relieve radiation induced xerostomia: a feasibility study. Acupuncture in Medicine,.2009; 27(3):109-113.

[171] Yang H Y, Wang J D, Lo T C, et al. Increased Mortality Risk for Cancers of the Kidney and Other Urinary Organs among Chinese Herbalists. Journal of Epidemiology.2009;19(1):17-23.

[172] WU Huan-lin, XU Dan-ping, LUO Wen-jie,et al. Retrospective cohort study of improving the prognosis of patients after coronary artery bypass grafting by justing-spleen-treating-heart.Jilin Journal of Traditional Chinese Medicine.Jan 2009;29(1):27-29. (in Chinese)

[173] WU Huan-lin, LUO Wen-jie, LIN Si-song, et al. Retrospective cohort study on justing-spleen-treating-heart

for PCI treatment of coronary heart disease. China Journal of Modern Medicine.Apr 2009;19(7):1220-1222. (in Chinese)

[174] GE Jian-zhong, YANG Yu-fei, XU Yun, et al. Clinical analysis of combined traditional Chinese and western medicine in treating recurrence and metastasis of elderly patients with colorectal cancer after radical resection. Chinese Journal of Gerontology.Jan 2009;29(1):73-76. (in Chinese)

[175] Ikue Watanabe, Shinichi Kuriyama, Masako Kakizaki，et al. Green tea and death from pneumonia in Japan: the Ohsaki cohort study.Am J Clin Nutr.Jul 2009;90:672-679.

[176] J. A. Olalde, et al. Clinical outcomes of diabetic foot management with Circulat. PHYTOTHERAPY RESEARCH 2008; 22: 1292-1298.

[177] Mohammad Heidary, et al. Effect of saffron on semen parameters of infertile men. Urology Journal 2008; 5:255-259.

[178] HUANG Xuerong, et al. Pharmaco epidemiological Study of Relationship between Puerarin Injection and Short-term Fever. Herald of Medicine 2008; 27(10): 1264-1266.

[179] LIANG Bi-yan, WU Yu, LUO Lin. Prospective Cohort Study on TCM to Protecting Relapse and Metastasis of Postoperative Colorectal Cancer.Chinese Journal of Information on TCM.2008;15(11):12-14.

[180] YANG Yu-fei, GE Jian-zhong, WU Yu, et al.Cohort Study on the Effect of a Combined Treatment of Traditional Chinese Medicine and Western Medicine on the

Relapse and Metastasis of 222 Patients with Stage II and IIIColorectal Cancer after Radical Operation. Chin J Integr Med 2008 Dec;14(4):251-256.

[181] S Jena, CM Witt, B Brinkhaus, K Wegscheider, et al. Acupuncture in patients with headache. Cephalalgia. 2008; 28(9):969-979.

[182] Jean-Francois Dartigues, et al. Vasodilators and nootropics as predictors of dementia and mortality in the PAQUID cohort JAGS 2007; 55: 395-399.

[183] Sun Chang. 79 cases of scapulohumeral periarthritis treated with acupuncture. Journal of New Chinese Medicine.2007.39(12):68-69.

[184] Lim K S, Tang M B Y, Goon A T J, et al. The role of topical traditional chinese medicaments as contact sensitisers in chronic venous leg ulcer patients. Ann Acad Med Singapore.2007; 36(11):942-946.

[185] Can-Lan Sun, Jian-Min Yuan, Woon Puay Koh，et al. Green tea and black tea consumption in relation to colorectal cancer risk: the Singapore Chinese Health Study. Carcinogenesis.Aug 2014;28(10):2143-2148.

[186] Farin Kamangar,Yu-Tang Gao,Xiao-Ou Shu，et al. Ginseng Intake and Gastric Cancer Risk in the Shanghai Women’s Health Study Cohort. Cancer Epidemiol Biomarkers Prev. Mar 2007;16(3):629-630.

[187] Li Guangxi, Liu zhiguo, Nie wenting, et al. Study on the relationship between Xiaochuan Ointment in the treatment of skin lesions caused by bronchial Asthma and its Clinical efficacy. China's Naturopathy.2006;14(10):40-42.

[188] Hull S K, Page C P, Skinner B D, et al. Exploring outcomes associated with acupuncture. The Journal of Alternative and Complementary Medicine. 2006;12(3):247-254.

[189] LUO Lin , YANG Yu-fei , LI Pei-hong, et al. Cohort Study on Fuzheng Capsule and Quxie Capsule in Reducing Relapse and Metastasis of Cancer in Patients with Stage Ⅱ and Ⅲ Colorectal Carcinoma after Operation.CJITWM.Aug 2006;26(8):677-680. (in Chinese)

[190] JOSEPH T.F.LAU, P.C.LEUNG, E.L.Y.WONG, et al.The Use of an Herbal Formula by Hospital Care Workers During the Severe Acute Respiratory Syndrome Epidemic in Hong Kong to Prevent Severe Acute Respiratory Syndrome Transmission, Relieve Influenza-Related Symptoms, and Improve Quality of Life: A Prospective Cohort Study. The Journal of Alternative and Complementary Medicine.2005;1(11): 49–55.

[191] Inoue M, Hojo T, Yano T, et al. Electroacupuncture direct to spinal nerves as an alternative to selective spinal nerve block in patients with radicular sciatica-a cohort study. Acupuncture in Medicine.2005;23(1):27-30.

[192] YUAN Yue-bin,WANG Zhong-qian,HU Xue-xin,et al.Cohort Study of Effects of Dinking Green Tea on Liver Diseases.Practical Preventive Medicine.Oct 2005;12(5):1016-1018.（in Chinese）

[193] Li Ping,Liu Baoyan,Weng Weiliang, et al. Influence of therapy integrated Chinese and western medicine on pulmonary inflammation of SARS. Journal of Beijing University of Traditional Chinese Medicine.Jul 2005; 28(4):55-57. (in Chinese)

[194] Jonas Persson, et al. The memory-enhancing effects of Ginseng and Ginkgo biloba in healthy volunteers. Psychopharmacology 2004; 172:430–434.

[195] NONG Yibing, et al. Constructing a Cox Proportional Hazard Regression Model of Prognosis Factors of Acute Myocardial Infarction by Retrospective Cohort Study. Chinese Journal of Integrated Chinese and Western Medicine 2004; 24(9): 781-784.

[196] Habs, M. Prospective, Comparative Cohort Studies and Their Contribution to the Benefit Assessments of Therapeutic Options: Heart Failure Treatment with and without Hawthorn Special Extract WS® 1442. Forschende Komplementärmedizin Klass Naturheilkunde 2004; 11(1):36-39.

[197] Blossfeldt, P. Acupuncture for chronic neck pain - a cohort study in an NHS pain clinic. Acupuncture in Medicine.2004;22(3):146-151.

[198] LIU Bao-yan,LI Ping,WENG Wei-liang,et al. Effect of early intervention with integrated Chinese and Western Medicine on pulmonary inflammation in SARS.Tianjin Journal of Traditional Chinese Medicine.Aug 2004; 21(4):268-271.(in Chinese)

[199] Stellon, A. Acupuncture in patients with valvular heart disease and prosthetic valves. Acupuncture in Medicine.2003;21(3):87-91.
