# Supplementary material for: A scoping review of cohort studies assessing traditional Chinese medicine interventions
Source: BMC Complement Med Ther. 2020 Nov 23;20:361. doi: 10.1186/s12906-020-03150-9 (PMC7684743; doi:10.1186/s12906-020-03150-9)
Supplement: Supplementary file 3 — Additional file 3. Reporting assessment of included TCM cohort studies using the STROBE-cohort checklist. [file 12906_2020_3150_MOESM3_ESM.docx]

Additional file 3 Reporting assessment of included TCM cohort studies using the STROBE-cohort checklist

*score “1” represents “sufficient reported”, “2” for “insufficient reported”, “3” for “unreported”, “4” for “not applicable”.

|  | **Item No** | **Recommendation** | **Score*** | | | |
| --- | --- | --- | --- | --- | --- | --- |
|  |  |  | 1 | 2 | 3 | 4 |
| **Title and abstract** | 1 | (a) Indicate the study’s design with a commonly used term in the title or the abstract | 41 | 97 | 61 | 0 |
|  |  | (b) Provide in the abstract an informative and balanced summary of what was done and what was found | 116 | 10 | 73 | 0 |
| **Introduction** | | |  |  |  |  |
| Background/rationale | 2 | Explain the scientific background and rationale for the investigation being reported | 158 | 12 | 29 | 0 |
| Objectives | 3 | State specific objectives, including any prespecified hypotheses | 94 | 23 | 82 | 0 |
| **Methods** | | |  |  |  |  |
| Study design | 4 | Present key elements of study design early in the paper | 66 | 49 | 84 | 0 |
| Setting | 5 | Describe the setting, locations, and relevant dates, including periods of recruitment, exposure, follow-up, and data collection | 59 | 1 | 3 | 136 |
| Participants | 6 | (a) Give the eligibility criteria, and the sources and methods of selection of participants. Describe methods of follow-up | 1 | 0 | 1 | 197 |
|  |  | (b) For matched studies, give matching criteria and number of exposed and unexposed | 38 | 0 | 3 | 158 |
| Variables | 7 | Clearly define all outcomes, exposures, predictors, potential confounders, and effect modifiers. Give diagnostic criteria, if applicable | 88 | 46 | 65 | 0 |
| Data sources/ measurement | 8 | For each variable of interest, give sources of data and details of methods of assessment (measurement). Describe comparability of assessment methods if there is more than one group | 199 | 0 | 0 | 0 |
| Bias | 9 | Describe any efforts to address potential sources of bias | 46 | 60 | 72 | 21 |
| Study size | 10 | Explain how the study size was arrived at | 53 | 10 | 135 | 1 |
| Quantitative variables | 11 | Explain how quantitative variables were handled in the analyses. If applicable, describe which groupings were chosen and why | 145 | 47 | 7 | 0 |
| Statistical methods | 12 | (a) Describe all statistical methods, including those used to control for confounding | 23 | 9 | 167 | 0 |
|  |  | (b) Describe any methods used to examine subgroups and interactions | 52 | 20 | 121 | 6 |
|  |  | (c) Explain how missing data were addressed | 139 | 41 | 16 | 3 |
|  |  | (d) If applicable, explain how loss to follow-up was addressed | 10 | 1 | 188 | 0 |
|  |  | (e) Describe any sensitivity analyses | 3 | 0 | 196 | 0 |
| **Results** | | |  |  |  |  |
| Participants | 13 | (a) Report numbers of individuals at each stage of study—eg numbers potentially eligible, examined for eligibility, confirmed eligible, included in the study, completing follow-up, and analysed | 8 | 0 | 191 | 0 |
|  |  | (b) Give reasons for non-participation at each stage | 16 | 0 | 183 | 0 |
|  |  | (c) Consider use of a flow diagram | 71 | 107 | 21 | 0 |
| Descriptive data | 14 | (a) Give characteristics of study participants (eg demographic, clinical, social) and information on exposures and potential confounders | 37 | 1 | 0 | 161 |
|  |  | (b) Indicate number of participants with missing data for each variable of interest | 14 | 0 | 185 | 0 |
|  |  | (c) Summarise follow-up time (eg, average and total amount) | 75 | 3 | 121 | 0 |
| Outcome data | 15 | Report numbers of outcome events or summary measures over time | 141 | 49 | 9 | 0 |
| Main results | 16 | (a) Give unadjusted estimates and, if applicable, confounder-adjusted estimates and their precision (eg, 95% confidence interval). Make clear which confounders were adjusted for and why they were included | 163 | 35 | 1 | 0 |
|  |  | (b) Report category boundaries when continuous variables were categorized | 28 | 3 | 0 | 168 |
|  |  | (c) If relevant, consider translating estimates of relative risk into absolute risk for a meaningful time period | 163 | 28 | 8 | 0 |
| Other analyses | 17 | Report other analyses done—eg analyses of subgroups and interactions, and sensitivity analyses | 145 | 44 | 10 | 0 |
| **Discussion** | | |  |  |  |  |
| Key results | 18 | Summarise key results with reference to study objectives | 152 | 47 | 0 | 0 |
| Limitations | 19 | Discuss limitations of the study, taking into account sources of potential bias or imprecision. Discuss both direction and magnitude of any potential bias | 138 | 53 | 8 | 0 |
| Interpretation | 20 | Give a cautious overall interpretation of results considering objectives, limitations, multiplicity of analyses, results from similar studies, and other relevant evidence | 114 | 78 | 7 | 0 |
| Generalisability | 21 | Discuss the generalisability (external validity) of the study results | 127 | 68 | 4 | 0 |
| **Other information** | | |  |  |  |  |
| Funding | 22 | Give the source of funding and the role of the funders for the present study and, if applicable, for the original study on which the present article is based | 178 | 5 | 16 | 0 |
